# Associations Between Healthy Behaviors and Persistently Favorable Self-Rated Health in a Longitudinal Population-Based Study in Switzerland

**DOI:** 10.1007/s11606-024-08739-1

**Published:** 2024-03-25

**Authors:** Mayssam Nehme, Stephanie Schrempft, Helene Baysson, Nick Pullen, Serguei Rouzinov, Silvia Stringhini, Speccio Study Group, Antoine Bal, Aminata Rosalie Bouhet, Paola D’ippolito, Roxane Dumont, Nacira El Merjani, Natalie Francioli, Severine Harnal, Stephane Joost, Gabriel Kathari, Julien Lamour, Andrea Jutta Loizeau, Elsa Lorthe, Chantal Martinez, Shannon Mecoullam, Caroline Pugin, Viviane Richard, Anshu Uppal, Jennifer Villers, María-Eugenia Zaballa, Idris Guessous

**Affiliations:** 1 Division of Primary Care Medicine, Geneva University Hospitals, Geneva, Switzerland; 2 Unit of Population Epidemiology, Division of Primary Care Medicine, Geneva University Hospitals, Geneva, Switzerland; 3 University Center for General Medicine and Public Health, University of Lausanne, Lausanne, Switzerland; 4 School of Population and Public Health and Edwin S.H, Leong Centre for Healthy Aging, Faculty of Medicine, University of British Columbia, Vancouver, Canada; 5 Faculty of Medicine, University of Geneva, Geneva, Switzerland

## Abstract

**Background:**

Self-rated health is a subjective yet valuable indicator of overall health status, influenced by various factors including physical, psychological, and socio-economic elements. Self-rated health could be telling and used by primary care physicians to evaluate overall present and predictive health.

**Design:**

This study investigates the longitudinal evolution of self-rated health in Switzerland during the COVID-19 pandemic, focusing on the association of persistently favorable self-rated health with various predictors.

**Participants:**

This study based on the Specchio cohort, a population-based digital study in Geneva Switzerland, involved participants completing questionnaires from 2021 to 2023.

**Main Measures:**

Self-rated health was assessed alongside factors like physical and mental health, socio-economic status, and lifestyle behaviors.

**Key Results:**

The study included 7006 participants in 2021, and 3888 participants who answered all three follow-ups (2021, 2022, and 2023). At baseline, 34.9% of individuals reported very good, 54.6% reported good, 9.6% reported average, and 1.0% reported poor to very poor self-rated health. Overall, 29.1% had a worsening in their self-rated health between 2021 and 2023. A subset of participants (12.1%) maintained very good self-rated health throughout, demonstrating persistently favorable self-rated health during the COVID-19 pandemic. Positive health behaviors were associated with persistently favorable self-rated health (exercise aOR 1.13 [1.03–1.24]; healthy diet aOR 2.14 [1.70–2.68]; less screen time aOR 1.28 [1.03–1.58]; and better sleep quality aOR 2.48 [2.02–3.04]). Mental health and social support also played significant roles.

**Conclusion:**

The study underscores the significance of healthy lifestyle choices and social support in maintaining favorable self-rated health, particularly during challenging times like the COVID-19 pandemic. Primary care physicians should focus on promoting these factors, integrating these actions in their routine consultations, and advising patients to undertake in socially engaging activities to improve overall health perceptions and outcomes.

**Supplementary Information:**

The online version contains supplementary material available at 10.1007/s11606-024-08739-1.

## BACKGROUND

Self-rated health is the individual’s own perception of his or her health in a holistic and broad way, and it is a valuable proxy of health status in general.^1^ It is usually assessed by answering one single question, and can be influenced by several physical, psychological, and socio-economic factors.^2^ Despite its subjective nature, self-rated health has been associated with a full array of illnesses, mortality, morbidity, functional impairment, hospitalizations, and even symptoms of disease not yet diagnosed.^3–6^ Studies have also shown associations between self-rated health and circulating cytokines,^7,8^ changes in hormonal levels,^9^ and immunity.^10^ Self-rated health is better interpreted when it is measured as a dynamic evaluation,^1^ on a trajectory in a longitudinal approach. In a large longitudinal analysis,^2^ compared to individuals with excellent self-rated health, individuals with good self-rated health had 20% greater mortality risk, and individuals with poor self-rated health had a 700% greater mortality risk.^2^

Determinants of self-rated health include cultural factors,^11^ age,^12^ and socio-economic factors. There is evidence that the association between self-rated health and mortality can differ between regions,^2^ socio-economic status, and sex.^2^ Poor self-rated health seems more predictive of mortality in men than in women,^1,13^ and in individuals with a lower socio-economic status.^2^ Different levels of education have been associated with significant variations in biomarkers (metabolic, cardiovascular, inflammatory, and organ function) within the same category of self-rated health.^14^ Similarly, lower education is associated with poorer self-rated health,^15^ and the same applies for lower income levels.^15^ Physical activity, better sleep quality, and sleep duration have also been associated with better health perception.^16^

Resilience is a concept that describes a stable trajectory of healthy functioning after a highly adverse event,^17^ and the capability of an individual to overcome stress.^18^ Several definitions exist for the concept of resilience, a complicated interaction of determinants (risk factors and protective factors) which leads to a positive adaptation after stressful situations. The association between self-rated health and resilience shows a bidirectional relationship seen across age groups.^19–22^ Self-rated health and resilience have also been linked to higher life satisfaction.^23–26^

Early in the COVID-19 pandemic, some studies showed that self-rated health was worsening, partly due to non-pharmaceutical interventions (NPIs) used to mitigate the spread of COVID-19, and partly due to worsening mental health in general, and increasing health inequalities.^27–29^ Additionally, SARS-CoV-2 infection was directly linked to post-COVID condition and worsening health outcomes in patients who suffered from post-COVID symptoms.^30,31^ Today, it is important to look back at the COVID-19 pandemic with lessons learned and try to understand who were the individuals who had a consistently positive perception of their self-rated health.

By understanding who rated their health favorably throughout the pandemic, there might be some insight as to which predictors were particularly associated with this group of individuals, and advice for primary care physicians in their practice. In this paper, we look at the longitudinal evolution of self-rated health in the general population in Switzerland and describe the association of persistently favorable self-rated health with predictors including age, sex, socio-economic status, and physical and mental health.

## METHODS

The Specchio cohort is a population-based digital study launched in December 2020.^32^ Initially, the study recruited participants during the COVID-19 pandemic for seroprevalence studies. Serosurvey participants were randomly selected from population registries and from the Bus Santé population-based study.^33^ The study was approved by the Cantonal Research Ethics Commission of Geneva, Switzerland (project number 2020-00881).

Participants completed a questionnaire at baseline, and regular follow-up questionnaires about general health as part of the Specchio cohort study. The questionnaires were administered in March 2021, March 2022, and March 2023. Follow-up questionnaires included questions about self-rated health, symptoms, new disease or health events, mental health, and questions on behavior (smoking, alcohol, physical activity). Participants were also invited to complete a more comprehensive behavioral questionnaire (diet, exercise, sleep habits, screen time) in May 2022.

Self-rated health was assessed by asking the question “How do you evaluate your current health” with answers 0 “very good,” 1 “good,” 2 “average,” 3 “poor,” and 4 “very poor.” Persistently favorable self-rated health was defined as self-rated health reported as very good over three data points (2021, 2022, and 2023), during the COVID-19 pandemic.

Exercise was defined as physical activity requiring considerable effort (i.e., jogging, biking, swimming, tennis, gymnastics, fitness); walking/fast walking or gardening was not considered an exercise. Screen time was defined as 0 “screen time of 2 hours or more for leisure purposes per day” and 1 “screen time of less than 2 hours for leisure purposes per day.” This was based on the recommendations of having 2 h or less of screen time per day. Questions about mental health and overall well-being included sleep quality, sleep time, and difficulty sleeping, feelings of isolation, the Oslo score for social support,^34^ and the WHO well-being index.^35^

Statistical analysis was done using STATA version 15.1 and R studio version 4.3.1. Descriptive analyses were used to evaluate the prevalence of good, very good, average, poor, and very poor self-rated health at the different time points. A generalized linear model was used to evaluate the evolution of self-rated health between 2021, 2022, and 2023. Stratification of the evolution of self-rated health was conducted by socio-economic determinants and health behaviors.

Logistic regression models were used to evaluate the association between persistently favorable self-rated health and the following predictors: age groups, sex, education, work situation, profession, living status, household income, pre-existing comorbidities, and pre-existing mental health condition in univariate analyses and multivariable analyses adjusted for the same factors. The associations of persistently favorable self-rated health with exercise, diet, screen habits, and sleep habits and change in habits were also evaluated using logistic regression models in univariate analyses and multivariable analyses adjusted for age, sex, education, work situation, profession, living status, household income, pre-existing medical comorbidities, pre-existing mental health condition, alcohol, smoking, social support, and the WHO well-being index. Interaction between mental health status based on the WHO well-being index “>50 lower risk of depression; 29-50 screening diagnosis of depression; ≤ 28 higher risk of depression” and age, physical activity, healthy diet, screen time, and sleep quality was evaluated. Interaction between social support as defined by the Oslo score “High social support; moderate social support; poor social support” and age, physical activity, healthy diet, screen time, and sleep quality was evaluated.

## RESULTS

Overall, *n* = 7006 participants were included in 2021, *n* = 6705 in 2022, and *n* = 5526 in 2023. More specifically, *n* = 3888 participants answered all three questionnaires, mean age 52.9 (standard deviation 12.9 years); 58.4% were women, 83.4% were Swiss nationals, 65.3% had a tertiary level of education, 56.6% had a middle-income status, and 63.6% were salaried (distributed between lower grade, higher grade while collar workers and professional manager categories). Out of participants, 23.0% had pre-existing comorbidities, and 2.0% had a pre-existing mental health condition. Table 1 shows the characteristics of participants at all time points. Table S1 shows the characteristics of both participants and non-participants.

**Table 1:** Baseline Characteristics of Participants at All Three Time Points

	2021 (*n* = 7006)	2022 (*n* = 6704)	2023 (*n* = 5519)	Participants to all 3 follow-ups (*n* = 3888)
	*N* (%)	*N* (%)	*N* (%)	*N* (%)
Age groups				
Under 25	241 (3.4)	164 (2.4)	134 (2.4)	61 (1.6)
Between 25 and 39	1395 (19.9)	1162 (17.3)	918 (16.6)	545 (14.0)
Between 40 and 64	4357 (62.2)	4237 (63.2)	3473 (62.9)	2506 (64.5)
65 and above	1013 (14.5)	1141 (17.0)	994 (18.0)	776 (20.0)
Sex				
Male	2942 (42)	2728 (40.7)	2238 (40.6)	1604 (41.3)
Female	4036 (57.6)	3955 (59)	3262 (59.1)	2271 (58.4)
Other	28 (0.4)	21 (0.3)	19 (0.3)	13 (0.3)
Education				
Primary	249 (3.6)	258 (3.8)	179 (3.2)	112 (2.9)
Secondary	2207 (31.5)	2105 (31.4)	1698 (30.8)	1234 (31.7)
Tertiary	4545 (64.9)	4327 (64.6)	3634 (65.9)	2538 (65.3)
Other	5 (0.1)	13 (0.2)	6 (0.1)	4 (0.1)
Work situation				
Salaried	4765 (68)	4392 (65.5)	3533 (64)	2474 (63.6)
Freelance/sole trader	475 (6.8)	459 (6.8)	386 (7)	287 (7.4)
Retired	1058 (15.1)	1207 (18)	1057 (19.2)	807 (20.8)
Unemployed	165 (2.4)	151 (2.3)	135 (2.4)	75 (1.9)
Other economically inactive	542 (7.7)	494 (7.4)	406 (7.4)	244 (6.3)
Profession				
Blue collar workers	637 (9.5)	594 (9.3)	427 (8.1)	298 (7.7)
Lower grade white collar workers	1732 (26)	1701 (26.7)	1388 (26.4)	999 (25.7)
Higher grade white collar workers	1978 (29.6)	1841 (28.9)	1553 (29.5)	1116 (28.7)
Professional-managers	2235 (33.5)	2124 (33.3)	1792 (34.1)	51 (1.3)
Independent workers	90 (1.3)	121 (1.9)	100 (1.9)	1294 (33.3)
Nationality				
Swiss nationals	5544 (79.1)	5337 (79.6)	4473 (81.0)	3241 (83.4)
Non-Swiss nationals	1462 (20.9)	1366 (20.4)	1046 (19.0)	647 (16.6)
Living status				
With partner and kids	3119 (44.5)	2975 (44.4)	2381 (43.2)	127 (3.3)
With partner, without kids	1877 (26.8)	1861 (27.8)	1587 (28.8)	1594 (41)
Cohabitation	497 (7.1)	401 (6)	323 (5.9)	1410 (36.3)
Single parent	484 (6.9)	443 (6.6)	385 (7)	249 (6.4)
Single	1029 (14.7)	1021 (15.2)	841 (15.2)	635 (16.3)
Household income				
Low	1048 (15)	982 (14.7)	753 (13.7)	497 (12.8)
Middle	3768 (53.8)	3593 (53.6)	3044 (55.2)	2199 (56.6)
High	960 (13.7)	955 (14.3)	795 (14.4)	574 (14.8)
Don’t know/don’t wish to answer	1229 (17.5)	1168 (17.4)	924 (16.8)	618 (15.9)
Pre-existing comorbidities	1499 (21.4)	1520 (22.7)	1262 (22.9)	893 (23.0)
Pre-existing mental health condition	147 (2.1)	157 (2.3)	125 (2.3)	77 (2.0)

**Figure 1:**
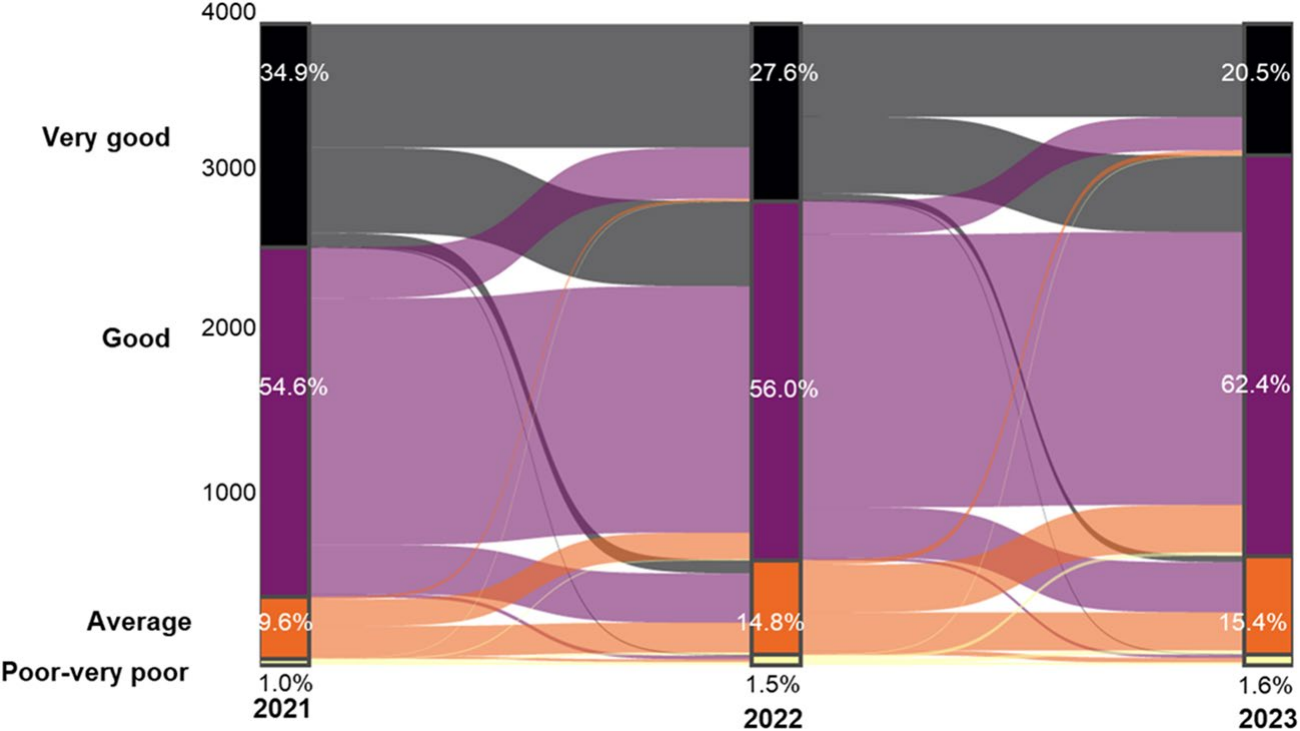
Longitudinal evolution of self-rated health between 2021 and 2023. Self-rated health was assessed by asking the question “How do you evaluate your current health” with answers 0 “very good,” 1 “good,” 2 “average,” 3 “poor,” and 4 “very poor.” Percentages show the prevalence of each category per year with the longitudinal evolution of each individual (*n* = 3888 answered all three follow-ups in 2021, 2022, and 2023).

At baseline, 34.9% of individuals reported very good, 54.6% reported good, 9.6% reported average, and 1.0% reported poor to very poor self-rated health. Overall, 4.5% reported an improvement in their self-rated health between 2021 and 2023, from good to very good, and 4.5% reported an improvement in their self-rated health from average to good. In parallel, 29.1% had a worsening in their self-rated health between 2021 and 2023, with 17.3% reporting a decrease in their self-rated health from very good to good, and 8.7% reporting a decrease from good to average. Figure 1 shows the evolution of self-rated health over time.

The distribution of self-rated health and its evolution did not significantly differ by age groups, although younger individuals tended to report more worsening than older individuals (32.8% in those younger than 25 versus 25.8% in those 65 years or older). Overall, 10.6% of women reported improvement in their self-rated health compared to 8.9% of men, and 30.2% of women reported worsening in their self-rated health compared to 27.7% of men. Table 2 shows the stratification of the evolution of self-rated health by groups.

**Table 2:** Evolution of Self-Rated Health Between 2021 and 2023 Stratified by Age Groups, Sex, Socio-economic Determinants and Comorbidities (*n* = 3888)*

	2021–2023			
	Improving (*n* = 389)	Stable (*n* = 2366)	Worsening (*n* = 1133)	*p*-value
	*N* (%)	*N* (%)	*N* (%)	
Age groups				0.091
Under 25	9 (14.7)	32 (52.5)	20 (32.8)	
Between 25 and 39	60 (11.0)	314 (57.6)	171 (31.4)	
Between 40 and 64	249 (9.9)	1515 (60.4)	742 (29.6)	
65 and above	71 (9.1)	505 (65.1)	200 (25.8)	
Sex				0.008
Male	144 (8.9)	1016 (63.3)	444 (27.7)	
Female	241 (10.6)	1345 (59.2)	685 (30.2)	
Other	4 (30.8)	5 (38.5(	4 (30.8)	
Education				0.214
Primary	13 (11.6)	72 (64.3)	27 (24.1)	
Secondary	106 (8.6)	753 (61.0)	375 (30.4)	
Tertiary	270 (10.6)	1537 (60.6)	731 (28.8)	
Other	0 (0)	4 (100.0)	0 (0)	
Work situation				0.240
Salaried	245 (9.9)	1491 (60.3)	738 (29.8)	
Freelance/sole trader	28 (9.8)	170 (59.2)	89 (31.0)	
Retired	73 (9.0)	521 (64.6)	213 (26.4)	
Unemployed	11 (14.7)	42 (56.0)	22 (29.3)	
Other economically inactive	32 (13.1)	141 (57.8)	71 (29.1)	
Profession				0.011
Blue collar workers	24 (8.0)	79 (26.5)	79 (7.2)	
Lower grade white collar workers	89 (8.9)	619 (62.0)	291 (29.1)	
Higher grade white collar workers	114 (10.2)	641 (57.4)	361 (32.3)	
Professional-managers	139 (10.7)	809 (62.5)	346 (26.7)	
Independent workers	6 (11.8)	23 (45.1)	22 (43.1)	
Nationality				0.616
Swiss nationals	139 (80.8)	2540 (83.6)	562 (83.0)	
Non-Swiss nationals	33 (19.2)	499 (16.4)	115 (17.0)	
Living situation				0.131
Single	79 (12.4)	360 (56.7)	196 (30.9)	
Single parent	28 (11.2)	153 (61.4)	68 (27.3)	
With partner and kids	144 (9.0)	966 (60.6)	484 (30.4)	
With partner, without kids	119 (9.7)	774 (63.3)	330 (27.0)	
Cohabitation	19 (10.2)	113 (60.4)	55 (29.4)	
Household income				0.041
Low	43 (8.6)	281 (56.5)	173 (34.8)	
Middle	225 (10.2)	1330 (60.5)	644 (29.3)	
High	62 (10.8)	356 (62.0)	156 (27.2)	
Don’t know/don’t wish to answer	59 (9.5)	399 (64.6)	160 (25.9)	
Pre-existing comorbidities				0.214
No	286 (9.5)	1835 (61.3)	874 (29.2)	
Yes	103 (11.5)	531 (59.5)	259 (29.0)	
Pre-existing mental health condition				0.120
No	376 (9.9)	2324 (61.0)	1111 (29.1)	
Yes	13 (16.9)	42 (54.5)	22 (28.6)	

*Individuals who answered all three follow-ups (2021, 2022, and 2023) were included in this analysis. Self-rated health was compared between 2021 (with answers 0 “very good,” 1 “good,” 2 “average,” 3 “poor,” 4 “very poor”) and 2023 (with answers 0 “very good,” 1 “good,” 2 “average,” 3 “poor,” 4 “very poor”). Improving was defined as any improvement in self-rated health (from very poor to poor or better; from poor to average or better; from good to very good). Stable was defined as stable self-rated health with no change in the answer between 2021 and 2023. Worsening was defined as worsening self-rated health (from very good to good or worse; from good to poor or worse; from poor to very poor)

Overall, *n* = 472 (12.1% of 3888 individuals) reported very good self-rated health in 2021, 2022, and 2023. These individuals were defined as having persistently favorable self-rated health, even when facing adverse events (COVID-19 pandemic). Tables 3 and 4 show persistently favorable self-rated health stratified by age groups, sex, education, profession, nationality, living status, household income, pre-existing comorbidities, pre-existing mental health conditions, health behavior, habits during the COVID-19 pandemic, and health events from 2021 to 2023. Individuals who consistently reported favorable self-rated health were less likely to be isolated, and more likely to have a moderate to high social support. Table S2 shows the association between persistently favorable self-rated health and the socio-economic determinants and non-modifiable risk factors.

**Table 3:** Stratification of Persistently Favorable Self-Rated Health by Socio-economic Determinants (*n* = 3888)*

	Persistently favorable self-rated health (*n* = 472)	Others (*n* = 3416)	Total (*n* = 3888)	*p*-value
	*N* (%)	*N* (%)	*N* (%)	
Age groups				0.526
Under 25	11 (2.3)	50 (1.5)	61 (1.6)	
Between 25 and 39	63 (13.3)	482 (14.1)	545 (14.0)	
Between 40 and 64	306 (64.8)	2200 (64.4)	2506 (64.5)	
65 and above	82 (19.5)	684 (20.0)	776 (20.0)	
Sex				0.025
Male	219 (46.4)	1385 (40.5)	1604 (41.3)	
Female	253 (53.6)	2018 (59.1)	2271 (58.4)	
Other	0 (0.0)	13 (0.4)	13 (0.3)	
Education				0.414
Primary	9 (1.9)	103 (3)	112 (2.9)	
Secondary	145 (30.7)	1089 (31.9)	1234 (31.7)	
Tertiary	318 (67.4)	2220 (65)	2538 (65.3)	
Other	0 (0.0)	4 (0.1)	4 (0.1)	
Work				0.464
Salaried	298 (63.3)	2176 (63.7)	2474 (63.6)	
Freelance/sole trader	43 (9.1)	244 (7.1)	287 (7.4)	
Retired	94 (20.0)	713 (20.9)	807 (20.8)	
Unemployed	6 (1.3)	69 (2.0)	75 (1.9)	
Other economically inactive	30 (6.4)	214 (6.3)	244 (6.3)	
Profession				0.101
Blue collar workers	30 (6.6)	268 (8.1)	298 (7.9)	
Lower grade white collar workers	119 (26.0)	880 (26.7)	999 (26.6)	
Higher grade white collar workers	121 (26.5)	995 (30.1)	1116 (29.7)	
Professional-managers	182 (39.8)	1112 (33.7)	1294 (34.4)	
Independent workers	5 (1.1)	46 (1.4)	51 (1.4)	
Nationality				0.549
Swiss nationals	398 (84.3)	573 (16.8)	3.241 (83.4)	
Non-Swiss nationals	74 (15.7)	2843 (83.2)	647 (16.8)	
Living status				0.595
Single	71 (15.0)	564 (16.5)	635 (16.3)	
Single parent	31 (6.6)	218 (6.4)	249 (6.4)	
With partner and kids	197 (41.7)	1397 (40.9)	1594 (41)	
With partner, without kids	144 (30.5)	1079 (31.6)	1223 (31.5)	
Cohabitation	29 (6.1)	158 (4.6)	187 (4.8)	
Household income				<0.001
Low	46 (9.7)	451 (13.2)	497 (12.8)	
Middle	260 (55.1)	1939 (56.8)	2199 (56.6)	
High	106 (22.5)	468 (13.7)	574 (14.8)	
Don’t know/don’t wish to answer	60 (12.7)	558 (16.3)	618 (15.9)	
Pre-existing comorbidities	41 (8.7)	852 (24.9)	893 (23.0)	<0.001
Pre-existing mental health condition	4 (0.8)	73 (2.1)	77 (2.0)	0.059

*Individuals who answered all three follow-ups were included in this analysis. Persistently favorable self-rated health was defined as individuals who reported very good self-rated health at all three follow-ups (2021, 2022, and 2023)

**Table 4:** Stratification of Persistently Favorable Self-Rated Health by Health Behavior and Events (*n* = 3549)*

	Persistently favorable self-rated health (*n* = 427)	Others (*n* = 3122)	Total (*n* = 3549)	*p*-value
	*N* (%)	*N* (%)	*N* (%)	
Health event	85 (19.9)	1084 (34.7)	1169 (32.9)	<0.001
Hospitalization	2 (0.5)	25 (0.8)	27 (0.8)	0.460
Life event				
Violence	4 (0.9)	79 (2.5)	83 (2.3)	0.041
Divorce	10 (2.3)	78 (2.5)	88 (2.5)	0.845
Job loss	9 (2.1)	56 (1.8)	65 (1.8)	0.650
Behavior				
Alcohol				0.005
Never	38 (8.9)	277 (8.9)	315 (8.9)	
Occasionally	128 (30)	1141 (36.5)	1269 (35.8)	
Once a week	61 (14.3)	342 (11)	403 (11.4)	
2–3 times per week	158 (37)	967 (31)	1125 (31.7)	
Daily	40 (9.4)	344 (11)	384 (10.8)	
Several times a day	2 (0.5)	51 (1.6)	53 (1.5)	
Smoking				0.063
Never smokers	254 (59.5)	1681 (53.8)	1935 (54.5)	
Former smokers	112 (26.2)	981 (31.4)	1093 (30.8)	
Current smokers	61 (14.3)	460 (14.7)	521 (14.7)	
Exercise				<0.001
Never	71 (16.6)	770 (24.7)	841 (23.7)	
Occasionally	77 (18)	804 (25.8)	881 (24.8)	
Once a week	101 (23.7)	714 (22.9)	815 (23)	
2–3 times a week	123 (28.8)	627 (20.1)	750 (21.1)	
3–4 times a week	36 (8.4)	132 (4.2)	168 (4.7)	
Daily	19 (4.4)	75 (2.4)	94 (2.6)	
Exercise during pandemic				<0.001
Decreased	14 (3.3)	277 (8.9)	291 (8.2)	
Slightly decreased	32 (7.5)	474 (15.2)	506 (14.3)	
Did not change	288 (67.4)	1628 (52.1)	1916 (54)	
Slightly increased	53 (12.4)	488 (15.6)	541 (15.2)	
Increased	40 (9.4)	255 (8.2)	295 (8.3)	
Healthy diet habits				<0.001
No	0 (0)	28 (0.9)	28 (0.8)	
Mostly no	5 (1.2)	236 (7.6)	241 (6.8)	
Yes	200 (46.8)	782 (25)	982 (27.7)	
Mostly yes	221 (51.8)	2032 (65.1)	2253 (63.5)	
Do not know	1 (0.2)	44 (1.4)	45 (1.3)	
Diet habits during pandemic				<0.001
Improved	26 (6.1)	159 (5.1)	185 (5.2)	
Slightly improved	35 (8.2)	384 (12.3)	419 (11.8)	
Worsened	1 (0.2)	37 (1.2)	38 (1.1)	
Slightly worsened	6 (1.4)	209 (6.7)	215 (6.1)	
Did not change	359 (84.1)	2333 (74.7)	2692 (75.9)	
Screen use				<0.001
Never	8 (1.9)	8 (0.3)	16 (0.5)	
< 1 h per day	79 (18.5)	457 (14.6)	536 (15.1)	
1 h to < 2 h per day	159 (37.2)	1116 (35.7)	1275 (35.9)	
2 h to < 4 h per day	143 (33.5)	1191 (38.1)	1334 (37.6)	
4 h to < 8 h per day	25 (5.9)	277 (8.9)	302 (8.5)	
≥ 8 h per day	13 (3)	73 (2.3)	86 (2.4)	
Screen habits during pandemic				<0.001
Increased	32 (7.5)	350 (11.2)	382 (10.8)	
Slightly increased	55 (12.9)	733 (23.5)	788 (22.2)	
Decreased	6 (1.4)	28 (0.9)	34 (1)	
Slightly decreased	6 (1.4)	94 (3)	100 (2.8)	
Did not change	328 (76.8)	1917 (61.4)	2245 (63.3)	
Sleep time				<0.001
< 5 h	2 (0.5)	50 (1.6)	52 (1.5)	
5–6 h	28 (6.6)	404 (12.9)	432 (12.2)	
6–7 h	135 (31.6)	1058 (33.9)	1193 (33.6)	
7–8 h	209 (48.9)	1267 (40.6)	1476 (41.6)	
8–9 h	50 (11.7)	303 (9.7)	353 (9.9)	
9–10 h	2 (0.5)	33 (1.1)	35 (1)	
≥ 10 h	1 (0.2)	7 (0.2)	8 (0.2)	
Sleep quality				<0.001
Very good	163 (38.2)	358 (11.5)	521 (14.7)	
Good	209 (48.9)	1939 (62.1)	2148 (60.5)	
Poor	55 (12.9)	753 (24.1)	808 (22.8)	
Very poor	0 (0.0)	72 (2.3)	72 (2.0)	
Sleep difficulties				<0.001
Never	168 (39.3)	567 (18.2)	735 (20.7)	
Sometimes	206 (48.2)	1744 (55.9)	1950 (54.9)	
Often	47 (11)	649 (20.8)	696 (19.6)	
All the time	6 (1.4)	162 (5.2)	168 (4.7)	
Sleep habits during pandemic				<0.001
Improved	8( 1.9)	45 (1.4)	53 (1.5)	
Slightly improved	7 (1.6)	111 (3.6)	118 (3.3)	
Worsened	4 (0.9)	151 (4.8)	155 (4.4)	
Slightly worsened	37 (8.7)	605 (19.4)	642 (18.1)	
Did not change	371 (86.9)	2210 (70.8)	2581 (72.7)	
Mental health and social support				
Isolated				<0.001
Never	352 (82.4)	1772 (56.8)	2124 (59.8)	
Rarely	57 (13.3)	774 (24.8)	831 (23.4)	
Sometimes	17 (4)	484 (15.5)	501 (14.1)	
Most of the time	1 (0.2)	80 (2.6)	81 (2.3)	
All the time	0 (0)	12 (0.4)	12 (0.3)	
Oslo score—interpretation				<0.001
≤ 8: Poor social support	42 (9.8)	543 (17.4)	585 (16.5)	
9–11: Moderate social support	224 (52.5)	1871 (59.9)	2095 (59)	
≥ 12: High social support	161 (37.7)	708 (22.7)	869 (24.5)	
WHO score—interpretation				<0.001
> 50: Lower risk of depression	416 (97.4)	2394 (76.7)	2810 (79.2)	
29–50: “Screening diagnosis” of depression	9 (2.1)	547 (17.5)	556 (15.7)	
≤ 28: Higher risk of depression	2 (0.5)	181 (5.8)	183 (5.2)	

*Individuals who answered all three follow-ups and the behavioral questionnaire were included in this analysis. Persistently favorable self-rated health was defined as individuals who reported very good self-rated health at all three follow-ups (2021, 2022, and 2023)

**Figure 2:**
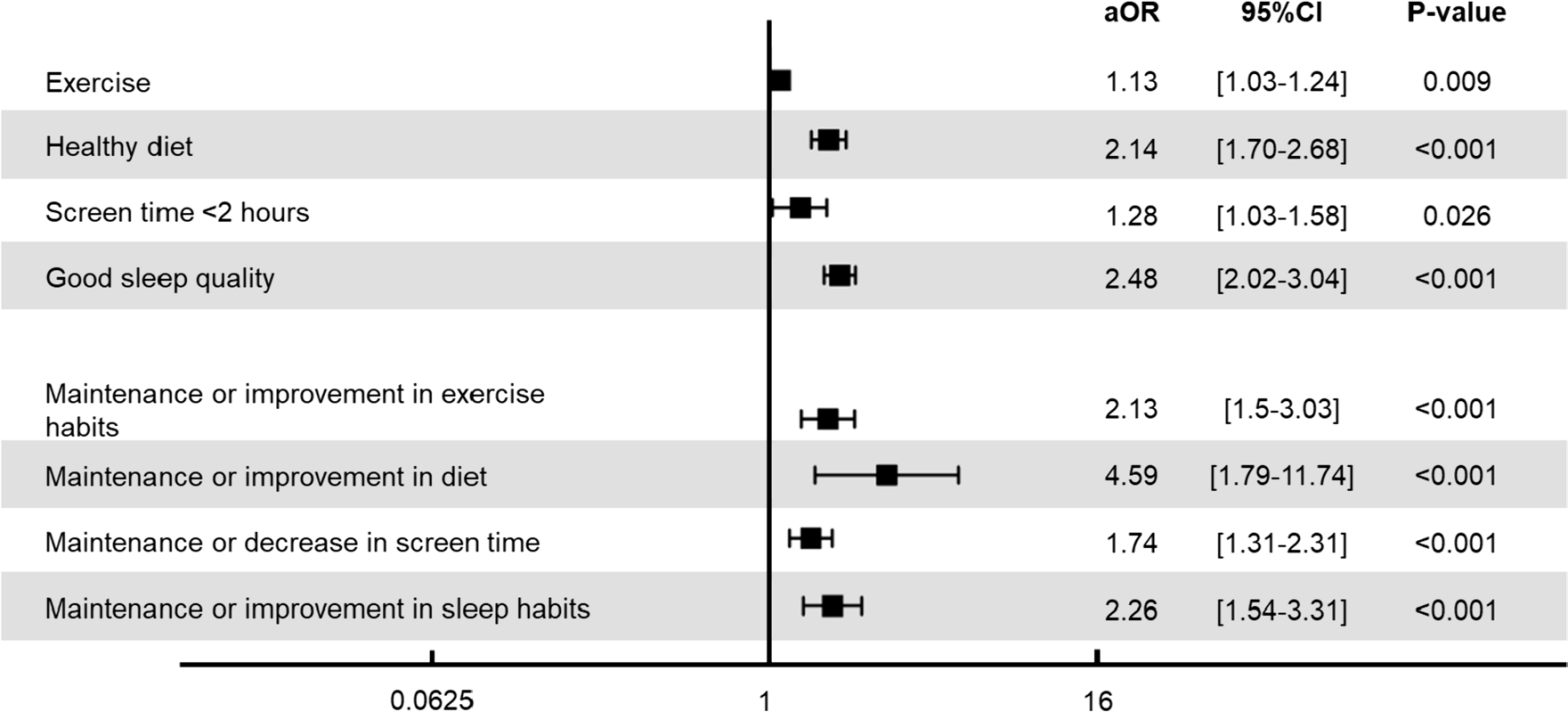
Association between health behaviors and persistently favorable self-rated health. OR, odds ratio; 95%CI, 95% confidence interval; aOR, adjusted odds ratio; adjusted for age groups, sex, education, work situation, profession, living status, household income, pre-existing comorbidities, pre-existing mental health condition, alcohol, smoking, social support, and mental health status. Exercise, healthy diet, sleep quality, and screen time were considered to be modifiable behaviors. The figure shows the association between these behaviors and changes in these behaviors with persistently favorable self-rated health.

Analyses evaluating the association between persistently favorable self-rated health and health behavior (exercise, diet, screen habits, and sleep) showed a positive association with exercise (aOR 1.13 [1.03–1.24]); healthy diet (aOR 2.14 [1.70–2.68]); less screen time (aOR 1.28 [1.03–1.58]); and sleep quality (aOR 2.48 [2.02–3.04]). Analyses evaluating the association between change in health behaviors and persistently favorable self-rated health (change in exercise, change in diet, change in screen habits, and change in sleep habits) showed a significant positive association with a maintenance or positive change in each of these habits. Figure 2 shows the association between persistently favorable self-rated health and health behaviors, as well as changes in health behavior, adjusted for socio-economic determinants, mental health, and social support. Considering the younger age group might have been more affected by mental health or social support factors, an interaction term between mental health and age was added to the analysis. Interaction between mental health and age showed the effect of age was conditional on mental health status. Similarly, interaction between social support and age showed the effect of age was conditional on social support. Interaction terms showed the effects of physical activity, healthy diet, screen time, and sleep quality were conditional on mental health. The same was true for social support. Table S3 shows the distribution of socio-economic determinants and health behaviors by age.

## DISCUSSION

While self-rated health worsened in the general population between 2021 and 2023, nearly one out of eight individuals reported a persistently favorable self-rated health throughout. These individuals might have factors contributing to their resilience in face of adverse events such as the COVID-19 pandemic. Persistently favorable self-rated health was more prevalent in individuals with maintained physical activity, healthy diet, reduced screen time, and good sleep quality. Inversely, the individuals who reported worsening in their self-rated health were also those who had the least physical activity including a decrease during the pandemic, the least healthy diets including worsening during the pandemic, more screen time exposure including an increase during the pandemic, and worse sleep quality including worsening during the pandemic.

Although we cannot exclude reverse causality, our results suggest that factors leading to a healthy lifestyle directly influence the perception of health. Self-rated health is associated with objective outcomes including mortality, cardiovascular disease, and other comorbidities.^2^ Studies early on in the pandemic showed that increasing risk of SARS-CoV-2 infection,^36^ adverse socio-economic factors,^37^ and the pandemic conditions in general^38,39^ were associated with worsening self-rated health. While these studies were very insightful, they lacked longitudinal population-based data showing evolution of self-rated health with time, and any insights into the individual factors that could be contributing to improved self-rated health. This current population-based study shows the evolution of self-rated health over a 3-year period, considering the impact of the COVID-19 pandemic that evolved with time, as well as individual behavior that contributed to persistently favorable self-rated health. Leading a healthy lifestyle, even through adverse times, could improve not only one’s perception of their own health, but also their health outcomes. The longitudinal aspect of this study, including changes in health behaviors, supports the association between positive health behaviors and persistently favorable self-rated health as an outcome, even though it is important to consider that this relationship could be bidirectional and partly influenced by factors such as physical and mental state of health.

The role of physical activity has already been studied in self-rated health.^40–42^ Physical activity was shown to be a potential mediator in the positive perception of aging and self-rated health;^41^ however, a combination of healthy behaviors including physical activity, diet, screen time use, and sleep quality has not been studied in their association with persistently favorable self-rated health. Our study showed that in addition to physical activity, a self-reported maintained healthy diet among other factors contributed to an overall favorable perception of self-rated health. Diet literacy^43,44^ and objective measurements would be interesting to explore.

The role of screen time in self-rated health has not been well studied to date. A pre-pandemic study in adolescents in 2011–2012 showed that 78% of adolescents who were active reported a very good to excellent self-rated health compared to 62% of adolescents who were not active, and 70% of adolescents exceeding screen time guidelines of 2 h per week reported a very good to excellent self-rated health compared to 77% of those who did not exceed the screen time guidelines.^45^ While physical activity was associated with improved self-rated health in that study, screen time was not. The results in our study showed that individuals who had persistently favorable self-rated health were those whose had screen time less than 2 h per day and whose screen time habits did not change during the pandemic.

Persistently favorable self-rated health was more prevalent among individuals who had better sleep quality, less sleep difficulties, and a sleep duration of 7–8 h on average. Their sleep habits did not worsen during the pandemic. Sleep is an important risk factor of mental health and self-rated health; a review of sleep duration showed that extremes of sleep duration (short or long) were associated with poor self-rated health.^46^ This was also true in a study of 689 young adults in 2017.^47^ Sleep is an important factor to look into as it influences physical and mental health and could potentially be a modifiable risk factor. Of note, sleep and mental health have a bidirectional relationship and any evaluation of sleep should consider the evaluation of mental health as well^48,49^. In our study, individuals who reported worsening sleep habits during the pandemic were most likely to report worsening in their self-rated health, thus suggesting an association between worse sleep and poorer self-rated health.

When looking into mental health factors, persistently favorable self-rated health was associated with better scores on the WHO well-being index and more social support. While it is important to identify individuals at risk of depression or other mental health outcomes, one approach could be to promote healthy behaviors as they contribute to the individual’s well-being and resilience. Considering that non-Swiss nationals could be more at risk of worsening self-rated health with less social support in Geneva, and the pandemic conditions prohibiting them from visiting their families abroad, association with nationality as a surrogate of having family abroad was evaluated. There were no differences found between Swiss nationals and non-Swiss nationals with regard to their self-rated health, even though non-Swiss nationals had significantly less social support as evidenced by the Oslo score (21.8% had poor social support compared to 15.4% of Swiss nationals). Overall, individuals who reported persistently favorable self-rated health were less likely to report feelings of isolation (82.8% reported never feeling isolated compared to 56.3%). Identification of isolation, increasing awareness campaigns against this phenomenon, and promoting social networks and community activities should be considered in the evaluation of general health, with special attention to immigrant populations.

When comparing self-rated health during the pandemic to pre-pandemic levels, results showed that self-rated health was potentially worsening in most demographic groups prior to the pandemic (fair or poor self-rated health increased from 13.4% in 1993 to 15.5% in 2021 in a study including 1.2 million adults).^50^ This in turn shows that self-rated health could worsen with time; however, the COVID-19 pandemic might have accelerated this deterioration. It is important to note that younger groups seemed more likely to report worsening self-rated health between 1993 and 2001 compared to the group of 65 years and above.^50^ This was also true in our study.

In our study, younger individuals had a tendency to report more worsening self-rated health with time (32.8% compared to 25.8% in the age group of 65 years and above). This difference in the evolution of self-rated health could be attributed to the perception of self-rated health that changes with age.^12^ A study showed that transcendence and purposefulness of well-being in life were correlated with age (even though the overall dimensions of well-being were not correlated with age)^51^. Other factors in our study that could explain the trend in younger individuals were worsening behavior during the pandemic including decrease in exercise habits, worsening dietary habits, increase in screen time exposure, worsening sleep quality, worsening mental health, and more feelings of isolation during the pandemic.

Women had a tendency to report worsening self-rated health (30.3% compared to 28.1% in men). This could be due to sex-related perceptions of health,^52^ as well as reporting bias.^53^ Women also reported less exercise, worsening dietary habits, increase in screen time exposure, worsening sleep quality, worsening mental health, and more feelings of isolation. Table S4 shows the distribution of these determinants by sex.

Limitations in this study include the self-reported nature of the symptoms and health behaviors, contributing to a potential reporting bias. However, this is mitigated by the fact that this study focused on self-rated health and the subjective nature of this measurement. Limitations also include potential reverse causality; however, the associations found are still significant and should be considered in the evaluation of patients and the population in general; future additional longitudinal data could help clarify these associations. Limitations also include a potential selection bias with a number of lost to follow-up in 2022 and 2023. Comparison between participants and non-participants showed differences in age, education, work situation, profession, living status, and household income. Socio-economic status could be a confounder enabling, for example, more well-off individuals to engage in healthy behaviors and improve their self-rated health. Adjustments for these socio-economic determinants and other factors were used to mitigate this limitation.

## CONCLUSION

While self-rated health worsened with time between 2021 and 2023, even in young otherwise healthy individuals who self-reported good to very good health initially, a group of individuals showed persistently favorable self-rated health and were more likely to engage in healthy behaviors. Lessons learned from resilience-exhibiting behavior are to promote healthy lifestyles and social networks. Primary care physicians should further stress on the importance of lifestyle behaviors including screen time, sleep habits, and socialization on top of the already known factors of physical activity and healthy diet. It is also important to take the time routinely to understand in detail the level and nature of physical activity that is being done, as well as what individuals might define as a healthy diet, a healthy amount of screen time, or healthy socialization. Primary care physicians should also pay special attention to immigrants lacking social support, and the younger population, often feeling more isolated and potentially suffering from more mental health problems. Promoting healthy behaviors, targeting potentially vulnerable groups, and emphasizing socially engaging activities might be tools to improve and maintain persistently favorable self-rated health in all age groups.

### Supplementary Information

Below is the link to the electronic supplementary material.

Supplementary file1 (DOCX 392 KB)

## Data Availability

Study data that underlie the results reported in this article can be made available to the scientific community after de-identification and upon submission of a data request application to the investigators study group via the corresponding author.
