# Supplementary material for: Associations Between Healthy Behaviors and Persistently Favorable Self-Rated Health in a Longitudinal Population-Based Study in Switzerland
Source: J Gen Intern Med. 2024 Mar 25;39(10):1828–38. doi: 10.1007/s11606-024-08739-1 (PMC11282021; doi:10.1007/s11606-024-08739-1)
Supplement: Supplementary file 1 — Supplementary file1 (DOCX 392 KB) [file 11606_2024_8739_MOESM1_ESM.docx]

**Associations between healthy behaviors and persistently favorable self-rated health in a longitudinal population-based study in Switzerland**

**Running title: healthy behaviors and persistently favorable self-rated health**

Mayssam Nehme^1,2*^ MD, Stephanie Schrempft^1,2^ PhD, Helene Baysson^1,2^ PhD, Nick Pullen^1,2^ PhD, Serguei Rouzinov^1,2^ PhD, Silvia Stringhini^1,2,3,4^ PhD, [Specchio study group], Idris Guessous^1,2,5^ MD, PhD

**Affiliations**

^1^ Division of Primary Care Medicine, Geneva University Hospitals, Geneva, Switzerland

^2^ Unit of Population Epidemiology, Division of Primary Care Medicine, Geneva University Hospitals, Geneva, Switzerland.

^3^ University Center for General Medicine and Public Health, University of Lausanne, Lausanne, Switzerland.

^4^ School of Population and Public Health and Edwin S.H. Leong Centre for Healthy Aging, Faculty of Medicine, University of British Columbia, Vancouver, Canada

^5^ Faculty of medicine, University of Geneva, Geneva, Switzerland

***Corresponding author: Mayssam Nehme** [Mayssam.nehme@hcuge.ch](mailto:Mayssam.nehme@hcuge.ch)

Co-authors:

[Stephanie.schrempft@hcuge.ch](mailto:Stephanie.schrempft@hcuge.ch)

[HeleneMariePierre.Baysson@hcuge.ch](mailto:HeleneMariePierre.Baysson@hcuge.ch)

[Nicholas.pullen@hcuge.ch](mailto:Nicholas.pullen@hcuge.ch)

[Serguei.rouzinov@hcuge.ch](mailto:Serguei.rouzinov@hcuge.ch)

[Silvia.stringhini@hcuge.ch](mailto:Silvia.stringhini@hcuge.ch)

[Idris.guessous@hcuge.ch](mailto:Idris.guessous@hcuge.ch)

**Word count: 2990**

Number of references: 47

Number of tables: 4

Number of figures: 2

**Conflicts of interest:** The authors report no conflict of interest.

**Funding:** This study was funded by the Swiss Federal Office of Public Health, the General Directorate of Health, the Private Foundation of the Geneva University Hospitals, the Swiss School of Public Health (Corona Immunitas Research Programme) and the Fondation des Grangettes.

**Specchio Study group:** Antoine Bal, Helene Baysson, Aminata Rosalie Bouhet, Paola D’ippolito, Roxane Dumont, Nacira El Merjani, Natalie Francioli, Idris Guessous, Severine Harnal, Stephane Joost, Gabriel Kathari, Julien Lamour, Andrea Jutta Loizeau, Elsa Lorthe, Chantal Martinez, Shannon Mecoullam, Mayssam Nehme, Caroline Pugin, Nick Pullen, Viviane Richard, Serguei Rouzinov, Stephanie Schrempft, Anshu Uppal, Silvia Stringhini, Jennifer Villers, María-Eugenia Zaballa

**Abstract**

**Background**

Self-rated health is a subjective yet valuable indicator of overall health status, influenced by various factors including physical, psychological, and socio-economic elements. Self-rated health could be telling and used by primary care physicians to evaluate overall present and predictive health.

**Design**

This study investigates the longitudinal evolution of self-rated health in Switzerland during the COVID-19 pandemic, focusing on the association of persistently favorable self-rated health with various predictors.

**Participants**

This study based on the Specchio cohort, a population-based digital study in Geneva Switzerland, involved participants completing questionnaires from 2021 to 2023.

**Main measures**

Self-rated health was assessed alongside factors like physical and mental health, socio-economic status, and lifestyle behaviors.

**Key results**

The study included 7,006 participants in 2021, and 3,888 participants who answered all 3 follow-ups (2021, 2022 and 2023). At baseline, 34.9% of individuals reported very good, 54.6% reported good, 9.6% reported average and 1.0% reported poor to very poor self-rated health. Overall, 29.1% had a worsening in their self-rated health between 2021 and 2023. A subset of participants (12.1%) maintained very good self-rated health throughout, demonstrating persistently favorable self-rated health during the COVID-19 pandemic. Positive health behaviors were associated with persistently favorable self-rated health (exercise aOR 1.13 [1.03-1.24]; healthy diet aOR 2.14 [1.70-2.68]; less screen time aOR 1.28 [1.03-1.58]; and better sleep quality aOR 2.48 [2.02-3.04]). Mental health and social support also played significant roles.

**Conclusion**

The study underscores the significance of healthy lifestyle choices and social support in maintaining favorable self-rated health, particularly during challenging times like the COVID-19 pandemic. Primary care physicians should focus on promoting these factors, integrating these actions in their routine consultations, and advising patients to undertake in socially engaging activities to improve overall health perceptions and outcomes.

**Background**

Self-rated health is the individual’s own perception of his or her health in a holistic and broad way, and it is a valuable proxy of health status in general^1^. It is usually assessed by answering one single question, and can be influenced by several physical, psychological and socio-economic factors^2^. Despite its subjective nature, self-rated health has been associated with a full array of illnesses, mortality, morbidity, functional impairment, hospitalizations, and even symptoms of disease not yet diagnosed^3–6^. Studies have also shown associations between self-rated health and circulating cytokines^7,8^, changes in hormonal levels^9^, and immunity^10^. Self-rated health is better interpreted when it is measured as a dynamic evaluation^1^, on a trajectory in a longitudinal approach. In a large longitudinal analysis^2^, compared to individuals with excellent self-rated health, individuals with good self-rated health had 20% greater mortality risk, and individuals with poor self-rated health had a 700% greater mortality risk^2^.

Determinants of self-rated health include cultural factors^11^, age ^12^, and socio-economic factors. There is evidence that the association between self-rated health and mortality can differ between regions^2^, socio-economic status and sex^2^. Poor self-rated health seems more predictive of mortality in men than in women^1,13^, and in individuals with a lower socio-economic status^2^. Different levels of education have been associated with significant variations in biomarkers (metabolic, cardiovascular, inflammatory and organ function) within the same category of self-rated health^14^. Similarly, lower education is associated with poorer self-rated health^15^, and the same applies for lower income levels^15^. Physical activity, better sleep quality and sleep duration have also been associated with better health perception^16^.

Resilience is a concept that describes a stable trajectory of healthy functioning after a highly adverse event^17^, and the capability of an individual to overcome stress^18^. Several definitions exist for the concept of resilience, a complicated interaction of determinants (risk factors and protective factors) which leads to a positive adaptation after stressful situations. The association between self-rated health and resilience shows a bidirectional relationship seen across age groups^19–22^. Self-rated health and resilience have also been linked to higher life satisfaction^23–26^.

Early in the COVID-19 pandemic, some studies showed that self-rated health was worsening, partly due to non-pharmaceutical interventions (NPIs) used to mitigate the spread of COVID-19, and partly due to worsening mental health in general, and increasing health inequalities^27–29^. Additionally, SARS-CoV-2 infection was directly linked to post-COVID condition and worsening health outcomes in patients who suffered from post-COVID symptoms^30,31^. Today, it is important to look back at the COVID-19 pandemic with lessons learned and try to understand who were the individuals who had a consistently positive perception of their self-rated health.

By understanding who rated their health favorably throughout the pandemic, there might be some insight as to which predictors were particularly associated with this group of individuals, and advice for primary care physicians in their practice. In this paper, we look at the longitudinal evolution of self-rated health in the general population in Switzerland and describe the association of persistently favorable self-rated health with predictors including age, sex, socio-economic status, physical and mental health.

**Methods**

The Specchio cohort is a population-based digital study launched in December 2020^32^. Initially, the study recruited participants during the COVID-19 pandemic for seroprevalence studies. Serosurvey participants were randomly selected from population registries and from the Bus Santé population-based study^33^. The study was approved by the Cantonal Research Ethics Commission of Geneva, Switzerland (project number 2020-00881).

Participants completed a questionnaire at baseline, and regular follow-up questionnaires about general health as part of the Specchio cohort study. The questionnaires were administered in March 2021, March 2022 and March 2023. Follow-up questionnaires included questions about self-rated health, symptoms, new disease or health events, mental health and questions on behavior (smoking, alcohol, physical activity). Participants were also invited to complete a more comprehensive behavioral questionnaire (diet, exercise, sleep habits, screen time) in May 2022.

Self-rated health was assessed by asking the question “How do you evaluate your current health” with answers 0 “very good”, 1 “good”, 2 “average”, 3 “poor”, 4 “very poor”. Persistently favorable self-rated health was defined as self-rated health reported as very good over 3 data points (2021, 2022 and 2023), during the COVID-19 pandemic.

Exercise was defined as physical activity requiring considerable effort (i.e. jogging, biking, swimming, tennis, gymnastics, fitness), walking/fast walking or gardening were not considered as exercise. Screen time was defined as 0 “screen time of 2 hours or more for leisure purposes per day”, and 1 “screen time of less than 2 hours for leisure purposes per day”. This was based on the recommendations of having 2 hours or less of screen time per day. Questions about mental health and overall well-being included sleep quality, sleep time, and difficulty sleeping, feelings of isolation, the Oslo score for social support^34^, and the WHO well-being index^35^.

Statistical analysis was done using STATA version 15.1 and R studio version 4.3.1. Descriptive analyses were used to evaluate the prevalence of good, very good, average, poor, and very poor self-rated health at the different time points. A generalized linear model was used to evaluate the evolution of self-rated health between 2021, 2022, and 2023. Stratification of the evolution of self-rated health was conducted by socio-economic determinants and health behaviors.

Logistic regression models were used to evaluate the association between persistently favorable self-rated health and the following predictors: age groups, sex, education, work situation, profession, living status, household income, pre-existing comorbidities and pre-existing mental health condition in univariate analyses and multivariable analyses adjusted for the same factors. The associations of persistently favorable self-rated health with exercise, diet, screen habits, and sleep habits and change in habits were also evaluated using logistic regression models in univariate analyses and multivariable analyses adjusted for age, sex, education, work situation, profession, living status, household income, pre-existing medical comorbidities, pre-existing mental health condition, alcohol, smoking, social support and the WHO well-being index. Interaction between mental health status based on the WHO well-being index “>50 lower risk of depression; 29-50 screening diagnosis of depression; ≤ 28 higher risk of depression” and age; physical activity, healthy diet, screen time, and sleep quality were evaluated. Interaction between social support as defined by the Oslo score “High social support; moderate social support; poor social support” and age; physical activity, healthy diet, screen time, and sleep quality were evaluated.

**Results**

Overall, n=7,006 participants were included in 2021, n=6,705 in 2022, and n=5,526 in 2023. More specifically, n=3’888 participants answered all three questionnaires, mean age 52.9 (standard deviation 12.9 years), 58.4% were women, 83.4% were Swiss nationals, 65.3% had a tertiary level of education, 56.6% had a middle-income status, and 63.6% were salaried (distributed between lower grade, higher grade while collar workers and professional managers categories). Out of participants, 23.0% had pre-existing comorbidities, and 2.0% had a pre-existing mental health condition. Table 1 shows the characteristics of participants at all time points. Table S1 shows the characteristics of both participants and non-participants.

At baseline, 34.9% of individuals reported very good, 54.6% reported good, 9.6% reported average and 1.0% reported poor to very poor self-rated health. Overall, 4.5% reported an improvement in their self-rated health between 2021 and 2023, from good to very good, and 4.5% reported an improvement in their self-rated health from average to good. In parallel, 29.1% had a worsening in their self-rated health between 2021 and 2023, with 17.3% reporting a decrease in their self-rated health from very good to good, and 8.7% reporting a decrease from good to average. Figure 1 shows the evolution of self-rated health over time.

The distribution of self-rated health and its evolution did not significantly differ by age groups, although younger individuals tended to report more worsening than older individuals (32.8% in those younger than 25 versus 25.8% in those 65 years or older). Overall, 10.6% of women reported improvement in their self-rated health compared to 8.9% of men, and 30.2% of women reported worsening in their self-rated health compared to 27.7% of men. Table 2 shows the stratification of the evolution of self-rated health by groups.

Overall, n=472 (12.1% of 3,888 individuals), reported very good self-rated health in 2021, 2022 and 2023. These individuals were defined as having persistently favorable self-rated health, even when facing adverse events (COVID-19 pandemic). Tables 3-4 show persistently favorable self-rated health stratified by age groups, sex, education, profession, nationality, living status, household income, pre-existing comorbidities, pre-existing mental health conditions, health behavior, habits during the COVID-19 pandemic, and health events from 2021-2023. Individuals who consistently reported favorable self-rated health were less likely to be isolated, and more likely to have a moderate to high social support. Table S2 shows the association between persistently favorable self-rated health and the socio-economic determinants and non-modifiable risk factors.

Analyses evaluating the association between persistently favorable self-rated health and health behavior (exercise, diet, screen habits, and sleep) showed a positive association with exercise aOR 1.13 [1.03-1.24]; healthy diet aOR 2.14 [1.70-2.68]; less screen time aOR 1.28 [1.03-1.58]; and sleep quality aOR 2.48 [2.02-3.04]. Analyses evaluating the association between change in health behaviors and persistently favorable self-rated health (change in exercise, change in diet, change in screen habits and change in sleep habits) showed a significant positive association with a maintenance or positive change in each of these habits. Figure 2 shows the association between persistently favorable self-rated health and health behaviors, as well as changes in health behavior, adjusted for socio-economic determinants, mental health and social support. Considering the younger age group might have been more affected by mental health or social support factors, an interaction term between mental health and age was added to the analysis. Interaction between mental health and age showed the effect of age was conditional on mental health status. Similarly, interaction between social support and age showed the effect of age was conditional on social support. Interaction terms showed the effect of physical activity, healthy diet, screen time, and sleep quality were conditional on mental health. The same was true for social support. Table S3 shows the distribution of socio-economic determinants and health behaviors by age.

**Discussion**

While self-rated health worsened in the general population between 2021-2023, nearly one out of eight individuals reported a persistently favorable self-rated health throughout. These individuals might have factors contributing to their resilience in face of adverse events such as the COVID-19 pandemic. Persistently favorable self-rated health was more prevalent in individuals with maintained physical activity, healthy diet, reduced screen time, and good sleep quality. Inversely, the individuals who reported worsening in their self-rated health were also those who had the least physical activity including a decrease during the pandemic, the least healthy diets including worsening during the pandemic, more screen time exposure including an increase during the pandemic, and worse sleep quality including worsening during the pandemic.

Although we cannot exclude reverse causality, our results suggest that factors leading to a healthy lifestyle directly influence the perception of health. Self-rated health is associated with objective outcomes including mortality, cardiovascular disease, and other comorbidities^2^. Studies early on in the pandemic showed that increasing risk of SARS-CoV-2 infection^36^, adverse socio-economic factors^37^, and the pandemic conditions in general^38,39^ were associated with worsening self-rated health. While these studies were very insightful, they lacked longitudinal population-based data showing evolution of self-rated health with time, and any insights into the individual factors that could be contributing to improved self-rated health. This current population-based study shows the evolution of self-rated health over a three-year period, considering the impact of the COVID-19 pandemic that evolved with time, as well as individual behavior that contributed to persistently favorable self-rated health. Leading a healthy lifestyle, even through adverse times, could not only improve one’s perception of their own health, but also their health outcomes. The longitudinal aspect of this study, including changes in health behaviors support the association between positive health behaviors and persistently favorable self-rated health as an outcome, even though it is important to consider that this relationship could be bidirectional and partly influenced by factors such as physical and mental state of health.

The role of physical activity has already been studied in self-rated health^40–42^. Physical activity was shown to be a potential mediator in the positive perception of ageing and self-rated health^41^, however a combination of healthy behaviors including physical activity, diet, screen time use and sleep quality have not been studied in their association with persistently favorable self-rated health. Our study showed that in addition to physical activity, a self-reported maintained healthy diet among other factors contributed to an overall favorable perception of self-rated health. Diet literacy^43,44^ and objective measurements would be interesting to explore.

The role of screen time in self-rated health has not been well studied to date. A pre-pandemic study in adolescents in 2011-2012 showed that 78% of adolescents who were active reported a very good to excellent self-rated health compared to 62% of adolescents who were not active, and 70% of adolescents exceeding screen time guidelines of 2 hours per week reported a very good to excellent self-rated health compared to 77% of those who did not exceed the screen time guidelines^45^. While physical activity was associated with improved self-rated health in that study, screen time was not. The results in our study showed that individuals who had persistently favorable self-rated health were those whose had screen time less than 2 hours per day and whose screen time habits did not change during the pandemic.

Persistently favorable self-rated health was more prevalent among individuals who had better sleep quality, less sleep difficulties and a sleep duration of 7-8 hours on average. Their sleep habits did not worsen during the pandemic. Sleep is an important risk factor of mental health and self-rated health, a review of sleep duration showed that extremes of sleep duration (short or long) was associated with poor self-rated health^46^. This was also true in a study of 689 young adults in 2017^47^. Sleep is an important factor to look into as it influences physical and mental health and could potentially be a modifiable risk factor. Of note, sleep and mental health have a bidirectional relationship and any evaluation of sleep should consider the evaluation of mental health as well^48,49^. In our study, individuals who reported worsening sleep habits during the pandemic were most likely to report worsening in their self-rated health thus suggesting an association between worse sleep and poorer self-rated health.

When looking into mental health factors, persistently favorable self-rated health was associated with better scores on the WHO well-being index and more social support. While it is important to identify individuals at risk of depression or other mental health outcomes, one approach could be to promote healthy behaviors as they contribute to the individual’s well-being and resilience. Considering that non-Swiss nationals could be more at risk of worsening self-rated health with less social support in Geneva, and the pandemic conditions prohibiting them from visiting their families abroad, association with nationality as a surrogate of having family abroad was evaluated. There were no differences found between Swiss nationals and non-Swiss nationals with regards to their self-rated health, even though non-Swiss nationals had significantly less social support as evidenced by the Oslo score (21.8% had poor social support compared to 15.4% of Swiss nationals). Overall, individuals who reported persistently favorable self-rated health were less likely to report feelings of isolation (82.8% reported never feeling isolated compared to 56.3%). Identification of isolation, increasing awareness campaigns against this phenomenon and promoting social networks and community activities should be considered in the evaluation of general health, with special attention to immigrant populations.

When comparing self-rated health during the pandemic to pre-pandemic levels, results showed that self-rated health was potentially worsening in most demographic groups prior to the pandemic (fair or poor self-rated health increased from 13.4% in 1993 to 15.5% in 2021 in a study including 1.2 million adults) ^50^. This in turn shows that self-rated health could worsen with time, however the COVID-19 pandemic might have accelerated this deterioration. It is important to note that younger groups seemed more likely to report worsening self-rated health between 1993 and 2001 compared to the group of 65 years and above^50^. This was also true in our study.

In our study, younger individuals had a tendency to report more worsening self-rated health with time (32.8% compared to 25.8% in the age group of 65 years and above). This difference in the evolution of self-rated health could be attributed to the perception of self-rated health that changes with age^12^. A study showed that transcendence and purposefulness of well-being in life were correlated with age (even though the overall dimensions of well-being were not correlated with age)^51^. Other factors in our study that could explain the trend in younger individuals were worsening behavior during the pandemic including decrease in exercise habits, worsening dietary habits, increase in screen time exposure, worsening sleep quality, worsening mental health, and more feelings of isolation during the pandemic.

Women had a tendency to report worsening self-rated health (30.3% compared to 28.1% in men). This could be due to sex-related perceptions of health^52^, as well as reporting bias^53^. Women also reported less exercise, worsening dietary habits, increase in screen time exposure, worsening sleep quality, worsening mental health and more feelings of isolation. Table S4 shows the distribution of these determinants by sex.

Limitations in this study include the self-reported nature of the symptoms and health behaviors, contributing to a potential reporting bias. However, this is mitigated by the fact that this study focused on self-rated health and the subjective nature of this measurement. Limitations also include potential reverse causality, however the associations found are still significant and should be considered in the evaluation of patients and the population in general, future additional longitudinal data could help clarify these associations. Limitations also include a potential selection bias with a number of lost to follow-up in 2022 and 2023. Comparison between participants and non-participants showed differences in age, education, work situation, profession, living status and household income. Socioeconomic status could be a confounder enabling for example more well-off individuals to engage in healthy behaviors and improve their self-rated health. Adjustments for these socio-economic determinants and other factors were used to mitigate this limitation.

**Conclusion**

While self-rated health worsened with time between 2021 and 2023, even in young otherwise healthy individuals who self-reported good to very good health initially, a group of individuals showed persistently favorable self-rated health and were more likely to engage in healthy behaviors. Lessons learned from resilience-exhibiting behavior are to promote healthy lifestyles and social networks. Primary care physicians should further stress on the importance of lifestyle behaviors including screen time, sleep habits and socialization on top of the already known factors of physical activity and healthy diet. It is also important to take the time routinely to understand in detail the level and nature of physical activity that is being done, as well as what individuals might define as a healthy diet, a healthy amount of screen time or healthy socialization. Primary care physicians should also pay special attention to immigrants lacking social support, and the younger population, often feeling more isolated and potentially suffering from more mental health problems. Promoting healthy behaviors, targeting potentially vulnerable groups, and emphasizing socially engaging activities might be tools to improve and maintain persistently favorable self-rated health in all age groups.

Table 1. Baseline characteristics of participants at all three time points

|  |  | **2021**  **(n=7,006)** | **2022**  **(n=6,704)** | **2023**  **(n=5,519)** | **Participants to all 3 follow-ups**  **(n=3,888)** |
| --- | --- | --- | --- | --- | --- |
|  |  | N(%) | N(%) | N(%) | N(%) |
| **Age groups** |  |  |  |  |  |
| under 25 |  | 241(3.4) | 164(2.4) | 134(2.4) | 61(1.6) |
| Between 25 and 39 |  | 1,395(19.9) | 1,162(17.3) | 918(16.6) | 545(14.0) |
| between 40 and 64 |  | 4,357(62.2) | 4,237(63.2) | 3,473(62.9) | 2,506(64.5) |
| 65 and above |  | 1,013(14.5) | 1,141(17.0) | 994(18.0) | 776(20.0) |
| **Sex** |  |  |  |  |  |
| Male |  | 2,942(42) | 2,728(40.7) | 2,238(40.6) | 1,604(41.3) |
| Female |  | 4,036(57.6) | 3,955(59) | 3,262(59.1) | 2,271(58.4) |
| Other |  | 28(0.4) | 21(0.3) | 19(0.3) | 13(0.3) |
| **Education** |  |  |  |  |  |
| Primary |  | 249(3.6) | 258(3.8) | 179(3.2) | 112(2.9) |
| Secondary |  | 2,207(31.5) | 2,105(31.4) | 1,698(30.8) | 1,234(31.7) |
| Tertiary |  | 4,545(64.9) | 4,327(64.6) | 3,634(65.9) | 2,538(65.3) |
| Other |  | 5(0.1) | 13(0.2) | 6(0.1) | 4(0.1) |
| **Work situation** |  |  |  |  |  |
| Salaried |  | 4,765(68) | 4,392(65.5) | 3,533(64) | 2,474(63.6) |
| Freelance/sole trader |  | 475(6.8) | 459(6.8) | 386(7) | 287(7.4) |
| Retired |  | 1,058(15.1) | 1,207(18) | 1,057(19.2) | 807(20.8) |
| Unemployed |  | 165(2.4) | 151(2.3) | 135(2.4) | 75(1.9) |
| Other economically inactive |  | 542(7.7) | 494(7.4) | 406(7.4) | 244(6.3) |
| **Profession** |  |  |  |  |  |
| Blue collar workers |  | 637(9.5) | 594(9.3) | 427(8.1) | 298(7.7) |
| Lower grade white collar workers |  | 1,732(26) | 1,701(26.7) | 1,388(26.4) | 999(25.7) |
| Higher-grade white collar workers |  | 1,978(29.6) | 1,841(28.9) | 1,553(29.5) | 1,116(28.7) |
| Professional-Managers |  | 2,235(33.5) | 2,124(33.3) | 1,792(34.1) | 51(1.3) |
| Independent workers |  | 90(1.3) | 121(1.9) | 100(1.9) | 1,294(33.3) |
| **Nationality** |  |  |  |  |  |
| Swiss nationals |  | 5,544(79.1) | 5,337(79.6) | 4,473(81.0) | 3,241(83.4) |
| Non-Swiss nationals |  | 1,462(20.9) | 1,366(20.4) | 1,046(19.0) | 647(16.6) |
| **Living status** |  |  |  |  |  |
| With partner and kids |  | 3,119(44.5) | 2,975(44.4) | 2,381(43.2) | 127(3.3) |
| With partner, without kids |  | 1,877(26.8) | 1,861(27.8) | 1,587(28.8) | 1,594(41) |
| Cohabitation |  | 497(7.1) | 401(6) | 323(5.9) | 1,410(36.3) |
| Single parent |  | 484(6.9) | 443(6.6) | 385(7) | 249(6.4) |
| Single |  | 1,029(14.7) | 1,021(15.2) | 841(15.2) | 635(16.3) |
| **Household income** |  |  |  |  |  |
| Low |  | 1,048(15) | 982(14.7) | 753(13.7) | 497(12.8) |
| Middle |  | 3,768(53.8) | 3,593(53.6) | 3,044(55.2) | 2,199(56.6) |
| High |  | 960(13.7) | 955(14.3) | 795(14.4) | 574(14.8) |
| Don't know/don't wish to answer |  | 1,229(17.5) | 1,168(17.4) | 924(16.8) | 618(15.9) |
| **Pre-existing comorbidities** |  | 1,499(21.4) | 1,520(22.7) | 1,262(22.9) | 893 (23.0) |
| **Pre-existing mental health condition** |  | 147(2.1) | 157(2.3) | 125(2.3) | 77(2.0) |

Figure 1. Longitudinal evolution of self-rated health between 2021 and 2023


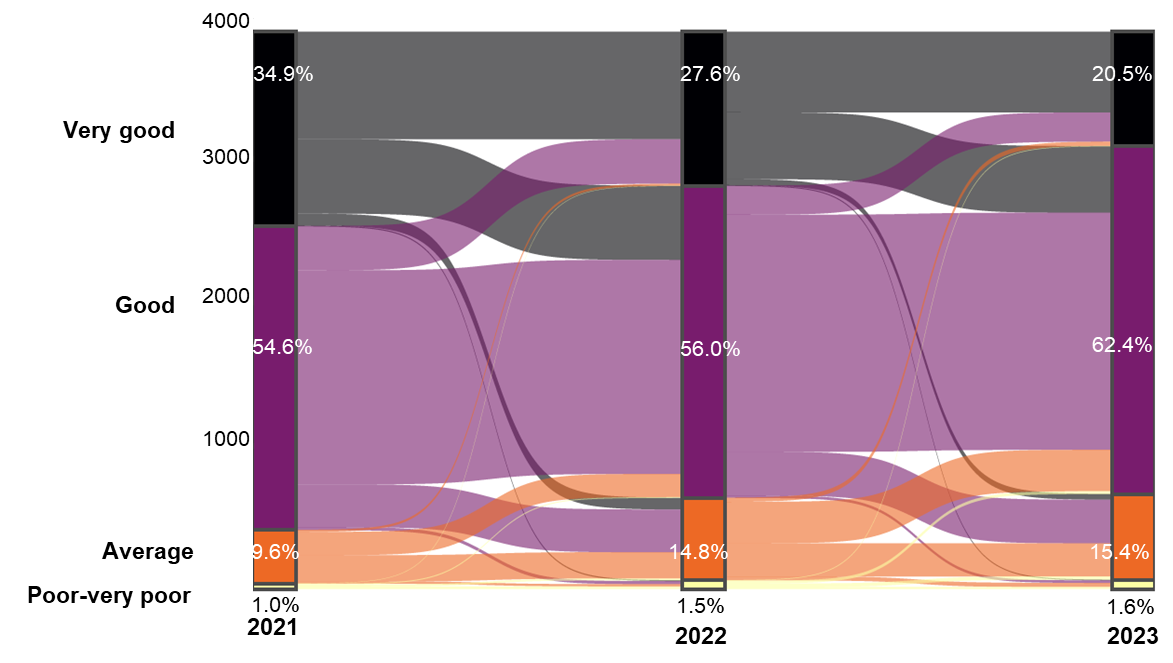


Self-rated health was assessed by asking the question “How do you evaluate your current health” with answers 0 “very good”, 1 “good”, 2 “average”, 3 “poor”, 4 “very poor”.

Percentages show the prevalence of each category per year with the longitudinal evolution of each individual (n=3,888 answered all three follow-ups in 2021, 2022 and 2023)


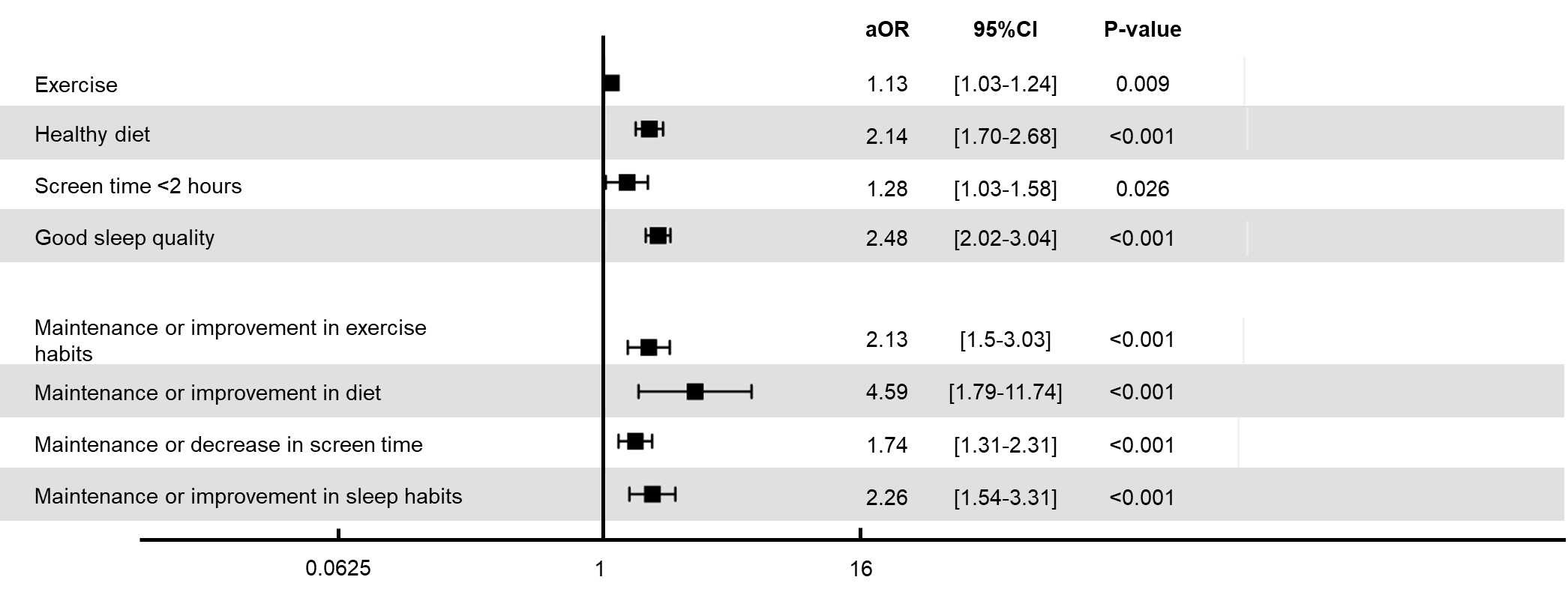
Figure 2. Association between health behaviors and persistently favorable self-rated health*

OR: Odds ratio

aOR: adjusted odds ratio, adjusted for age groups, sex, education, work situation, profession, living status, household income, pre-existing comorbidities, pre-existing mental health condition, alcohol, smoking, social support, and mental health status.

95%CI: 95% confidence interval

Exercise, healthy diet, sleep quality, screen time were considered to be modifiable behaviors. The figure shows the association between these behaviors and changes in these behaviors with persistently favorable self-rated health.

Table 2. Evolution of self-rated health between 2021-2023 stratified by age groups, sex, socio-economic determinants and co-morbidities (n=3,888)*

|  |  | **2021-2023** |  |  |
| --- | --- | --- | --- | --- |
|  | **Improving**  **(n=389)** | **Stable**  **(n=2,366)** | **Worsening**  **(n=1,133)** | **P-value** |
|  | **N(%)** | **N(%)** | **N(%)** |  |
| **Age groups** |  |  |  | 0.091 |
| under 25 | 9(14.7) | 32(52.5) | 20(32.8) |  |
| Between 25 and 39 | 60(11.0) | 314(57.6) | 171(31.4) |  |
| between 40 and 64 | 249(9.9) | 1,515(60.4) | 742(29.6) |  |
| 65 and above | 71(9.1) | 505(65.1) | 200(25.8) |  |
| **Sex** |  |  |  | 0.008 |
| Male | 144(8.9) | 1,016(63.3) | 444(27.7) |  |
| Female | 241(10.6) | 1,345(59.2) | 685(30.2) |  |
| Other | 4(30.8) | 5(38.5( | 4(30.8) |  |
| **Education** |  |  |  | 0.214 |
| Primary | 13(11.6) | 72(64.3) | 27(24.1) |  |
| Secondary | 106(8.6) | 753(61.0) | 375)30.4) |  |
| Tertiary | 270(10.6) | 1,537(60.6) | 731(28.8) |  |
| Other | 0(0) | 4(100.0) | 0(0) |  |
| **Work situation** |  |  |  | 0.240 |
| Salaried | 245(9.9) | 1,491(60.3) | 738(29.8) |  |
| Freelance/sole trader | 28(9.8) | 170(59.2) | 89(31.0) |  |
| Retired | 73(9.0) | 521(64.6) | 213(26.4) |  |
| Unemployed | 11(14.7) | 42(56.0) | 22(29.3) |  |
| Other economically inactive | 32(13.1) | 141(57.8) | 71(29.1) |  |
| **Profession** |  |  |  | 0.011 |
| Blue collar workers | 24(8.0) | 79(26.5) | 79(7.2) |  |
| Lower grade white collar workers | 89(8.9) | 619(62.0) | 291(29.1) |  |
| Higher-grade white collar workers | 114(10.2) | 641(57.4) | 361(32.3) |  |
| Professional-Managers | 139(10.7) | 809(62.5) | 346(26.7) |  |
| Independent workers | 6(11.8) | 23(45.1) | 22(43.1) |  |
| **Nationality** |  |  |  | 0.616 |
| Swiss nationals | 139(80.8) | 2,540(83.6) | 562(83.0) |  |
| Non-Swiss nationals | 33(19.2) | 499(16.4) | 115(17.0) |  |
| **Living situation** |  |  |  | 0.131 |
| Single | 79(12.4) | 360(56.7) | 196(30.9) |  |
| Single parent | 28(11.2) | 153(61.4) | 68(27.3) |  |
| With partner and kids | 144(9.0) | 966(60.6) | 484(30.4) |  |
| With partner, without kids | 119(9.7) | 774(63.3) | 330(27.0) |  |
| Cohabitation | 19(10.2) | 113(60.4) | 55(29.4) |  |
| **Household income** |  |  |  | 0.041 |
| Low | 43(8.6) | 281(56.5) | 173(34.8) |  |
| Middle | 225(10.2) | 1,330(60.5) | 644(29.3) |  |
| High | 62(10.8) | 356(62.0) | 156(27.2) |  |
| Don't know/don't wish to answer | 59(9.5) | 399(64.6) | 160(25.9) |  |
| **Pre-existing comorbidities** |  |  |  | 0.214 |
| No | 286(9.5) | 1,835(61.3) | 874(29.2) |  |
| Yes | 103(11.5) | 531(59.5) | 259(29.0) |  |
| **Pre-existing mental health condition** |  |  |  | 0.120 |
| No | 376(9.9) | 2,324(61.0) | 1,111(29.1) |  |
| Yes | 13(16.9) | 42(54.5) | 22(28.6) |  |

*Individuals who answered all three follow-ups (2021, 2022 and 2023) were included in this analysis. Self-rated health was compared between 2021 (with answers 0 “very good”, 1 “good”, 2 “average”, 3 “poor”, 4 “very poor”) and 2023 (with answers 0 “very good”, 1 “good”, 2 “average”, 3 “poor”, 4 “very poor”). Improving was defined as any improvement in self-rated health (from very poor to poor or better; from poor to average or better; from good to very good). Stable was defined as stable self-rated health with no change in the answer between 2021 and 2023. Worsening was defined as worsening self-rated health (from very good to good or worse; from good to poor or worse; from poor to very poor).

Table 3. Stratification of persistently favorable self-rated health by socio-economic determinants (n=3,888)*

|  | **Persistently favorable self-rated health (n=472)** | **Others (n=3,416)** | **Total (n=3,888)** | **P-value** |
| --- | --- | --- | --- | --- |
|  | N(%) | N(%) | N(%) |  |
| **Age groups** |  |  |  | 0.526 |
| under 25 | 11(2.3) | 50(1.5) | 61(1.6) |  |
| between 25 and 39 | 63(13.3) | 482(14.1) | 545(14.0) |  |
| between 40 and 64 | 306(64.8) | 2,200(64.4) | 2,506(64.5) |  |
| 65 and above | 82(19.5) | 684(20.0) | 776(20.0) |  |
| **Sex** |  |  |  | 0.025 |
| Male | 219(46.4) | 1,385(40.5) | 1,604(41.3) |  |
| Female | 253(53.6) | 2,018(59.1) | 2,271(58.4) |  |
| Other | 0(0.0) | 13(0.4) | 13(0.3) |  |
| **Education** |  |  |  | 0.414 |
| Primary | 9(1.9) | 103(3) | 112(2.9) |  |
| Secondary | 145(30.7) | 1,089(31.9) | 1,234(31.7) |  |
| Tertiary | 318(67.4) | 2,220(65) | 2,538(65.3) |  |
| Other | 0(0.0) | 4(0.1) | 4(0.1) |  |
| **Work** |  |  |  | 0.464 |
| Salaried | 298(63.3) | 2,176(63.7) | 2,474(63.6) |  |
| Freelance/sole trader | 43(9.1) | 244(7.1) | 287(7.4) |  |
| Retired | 94(20.0) | 713(20.9) | 807(20.8) |  |
| Unemployed | 6(1.3) | 69(2.0) | 75(1.9) |  |
| Other economically inactive | 30(6.4) | 214(6.3) | 244(6.3) |  |
| **Profession** |  |  |  | 0.101 |
| Blue collar workers | 30(6.6) | 268(8.1) | 298(7.9) |  |
| Lower grade white collar workers | 119(26.0) | 880(26.7) | 999(26.6) |  |
| Higher-grade white collar workers | 121(26.5) | 995(30.1) | 1,116(29.7) |  |
| Professional-Managers | 182(39.8) | 1,112(33.7) | 1,294(34.4) |  |
| Independent workers | 5(1.1) | 46(1.4) | 51(1.4) |  |
| **Nationality** |  |  |  | 0.549 |
| Swiss nationals | 398(84.3) | 573(16.8) | 3.241(83.4) |  |
| Non-Swiss nationals | 74(15.7) | 2,843(83.2) | 647(16.8) |  |
| **Living status** |  |  |  | 0.595 |
| Single | 71(15.0) | 564(16.5) | 635(16.3) |  |
| Single parent | 31(6.6) | 218(6.4) | 249(6.4) |  |
| With partner and kids | 197(41.7) | 1,397(40.9) | 1,594(41) |  |
| With partner, without kids | 144(30.5) | 1,079(31.6) | 1,223(31.5) |  |
| Cohabitation | 29(6.1) | 158(4.6) | 187(4.8) |  |
| **Household income** |  |  |  | <0.001 |
| Low | 46(9.7) | 451(13.2) | 497(12.8) |  |
| Middle | 260(55.1) | 1,939(56.8) | 2,199(56.6) |  |
| High | 106(22.5) | 468(13.7) | 574(14.8) |  |
| Don't know/don't wish to answer | 60(12.7) | 558(16.3) | 618(15.9) |  |
| Pre-existing Comorbidities | 41(8.7) | 852(24.9) | 893(23.0) | <0.001 |
| Pre-existing mental health condition | 4(0.8) | 73(2.1) | 77(2.0) | 0.059 |

*Individuals who answered all three follow-ups were included in this analysis. Persistently favorable self-rated health was defined as individuals who reported very good self-rated health at all three follow-ups (2021, 2022 and 2023)

Table 4. Stratification of persistently favorable self-rated health by health behavior and events (n=3,549)*

|  | **Persistently favorable self-rated health (n=427)** | **Others (n=3,122)** | **Total (n=3,549)** | P-value |
| --- | --- | --- | --- | --- |
|  | N(%) | N(%) | N(%) |  |
| **Health event** | 85(19.9) | 1,084(34.7) | 1,169(32.9) | <0.001 |
| Hospitalization | 2(0.5) | 25(0.8) | 27(0.8) | 0.460 |
| **Life event** |  |  |  |  |
| Violence | 4(0.9) | 79(2.5) | 83(2.3) | 0.041 |
| Divorce | 10(2.3) | 78(2.5) | 88(2.5) | 0.845 |
| Job loss | 9(2.1) | 56(1.8) | 65(1.8) | 0.650 |
| **Behavior** |  |  |  |  |
| **Alcohol** |  |  |  | 0.005 |
| Never | 38(8.9) | 277(8.9) | 315(8.9) |  |
| Occasionally | 128(30) | 1,141(36.5) | 1,269(35.8) |  |
| Once a week | 61(14.3) | 342(11) | 403(11.4) |  |
| 2-3 times per week | 158(37) | 967(31) | 1,125(31.7) |  |
| Daily | 40(9.4) | 344(11) | 384(10.8) |  |
| Several times a day | 2(0.5) | 51(1.6) | 53(1.5) |  |
| **Smoking** |  |  |  | 0.063 |
| Never smokers | 254(59.5) | 1,681(53.8) | 1,935(54.5) |  |
| Former smokers | 112(26.2) | 981(31.4) | 1,093(30.8) |  |
| Current smokers | 61(14.3) | 460(14.7) | 521(14.7) |  |
| **Exercise** |  |  |  | <0.001 |
| Never | 71(16.6) | 770(24.7) | 841(23.7) |  |
| Occasionally | 77(18) | 804(25.8) | 881(24.8) |  |
| Once a week | 101(23.7) | 714(22.9) | 815(23) |  |
| 2-3 times a week | 123(28.8) | 627(20.1) | 750(21.1) |  |
| 3-4 times a week | 36(8.4) | 132(4.2) | 168(4.7) |  |
| Daily | 19(4.4) | 75(2.4) | 94(2.6) |  |
| **Exercise during pandemic** |  |  |  | <0.001 |
| Decreased | 14(3.3) | 277(8.9) | 291(8.2) |  |
| Slightly decreased | 32(7.5) | 474(15.2) | 506(14.3) |  |
| Did not change | 288(67.4) | 1,628(52.1) | 1,916(54) |  |
| Slightly increased | 53(12.4) | 488(15.6) | 541(15.2) |  |
| Increased | 40(9.4) | 255(8.2) | 295(8.3) |  |
| **Healthy diet habits** |  |  |  | <0.001 |
| No | 0(0) | 28(0.9) | 28(0.8) |  |
| Mostly no | 5(1.2) | 236(7.6) | 241(6.8) |  |
| Yes | 200(46.8) | 782(25) | 982(27.7) |  |
| Mostly yes | 221(51.8) | 2,032(65.1) | 2,253(63.5) |  |
| Do not know | 1(0.2) | 44(1.4) | 45(1.3) |  |
| **Diet habits during pandemic** |  |  |  | <0.001 |
| Improved | 26(6.1) | 159(5.1) | 185(5.2) |  |
| Slighly improved | 35(8.2) | 384(12.3) | 419(11.8) |  |
| Worsened | 1(0.2) | 37(1.2) | 38(1.1) |  |
| Slightly worsened | 6(1.4) | 209(6.7) | 215(6.1) |  |
| Did not change | 359(84.1) | 2,333(74.7) | 2,692(75.9) |  |
| **Screen use** |  |  |  | <0.001 |
| Never | 8(1.9) | 8(0.3) | 16(0.5) |  |
| < 1 hour per day | 79(18.5) | 457(14.6) | 536(15.1) |  |
| 1 hour to <2 hours per day | 159(37.2) | 1,116(35.7) | 1,275(35.9) |  |
| 2 hours to <4 hours per day | 143(33.5) | 1,191(38.1) | 1,334(37.6) |  |
| 4 hours to <8 hours per day | 25(5.9) | 277(8.9) | 302(8.5) |  |
| ≥ 8 hours per day | 13(3) | 73(2.3) | 86(2.4) |  |
| **Screen habits during pandemic** |  |  |  | <0.001 |
| Increased | 32(7.5) | 350(11.2) | 382(10.8) |  |
| Slightly increased | 55(12.9) | 733(23.5) | 788(22.2) |  |
| Decreased | 6(1.4) | 28(0.9) | 34(1) |  |
| Slightly decreased | 6(1.4) | 94(3) | 100(2.8) |  |
| Did not change | 328(76.8) | 1,917(61.4) | 2,245(63.3) |  |
| **Sleep time** |  |  |  | <0.001 |
| < 5 hours | 2(0.5) | 50(1.6) | 52(1.5) |  |
| 5-6 hours | 28(6.6) | 404(12.9) | 432(12.2) |  |
| 6-7 hours | 135(31.6) | 1,058(33.9) | 1,193(33.6) |  |
| 7-8 hours | 209(48.9) | 1,267(40.6) | 1,476(41.6) |  |
| 8-9 hours | 50(11.7) | 303(9.7) | 353(9.9) |  |
| 9-10 hours | 2(0.5) | 33(1.1) | 35(1) |  |
| ≥ 10 hours | 1(0.2) | 7(0.2) | 8(0.2) |  |
| **Sleep quality** |  |  |  | <0.001 |
| Very good | 163(38.2) | 358(11.5) | 521(14.7) |  |
| Good | 209(48.9) | 1,939(62.1) | 2,148(60.5) |  |
| Poor | 55(12.9) | 753(24.1) | 808(22.8) |  |
| Very poor | 0(0.0) | 72(2.3) | 72(2.0) |  |
| **Sleep difficulties** |  |  |  | <0.001 |
| Never | 168(39.3) | 567(18.2) | 735(20.7) |  |
| Sometimes | 206(48.2) | 1,744(55.9) | 1,950(54.9) |  |
| Often | 47(11) | 649(20.8) | 696(19.6) |  |
| All the time | 6(1.4) | 162(5.2) | 168(4.7) |  |
| **Sleep habits during pandemic** |  |  |  | <0.001 |
| Improved | 8(1.9) | 45(1.4) | 53(1.5) |  |
| Slightly improved | 7(1.6) | 111(3.6) | 118(3.3) |  |
| Worsened | 4(0.9) | 151(4.8) | 155(4.4) |  |
| Slightly worsened | 37(8.7) | 605(19.4) | 642(18.1) |  |
| Did not change | 371(86.9) | 2,210(70.8) | 2,581(72.7) |  |
| **Mental health and social support** |  |  |  |  |
| **Isolated** |  |  |  | <0.001 |
| Never | 352(82.4) | 1,772(56.8) | 2,124(59.8) |  |
| Rarely | 57(13.3) | 774(24.8) | 831(23.4) |  |
| Sometimes | 17(4) | 484(15.5) | 501(14.1) |  |
| Most of the time | 1(0.2) | 80(2.6) | 81(2.3) |  |
| All the time | 0(0) | 12(0.4) | 12(0.3) |  |
| **Oslo score – interpretation** |  |  |  | <0.001 |
| ≤ 8: Poor social support | 42(9.8) | 543(17.4) | 585(16.5) |  |
| 9-11: Moderate social support | 224(52.5) | 1,871(59.9) | 2,095(59) |  |
| ≥ 12: High social support | 161(37.7) | 708(22.7) | 869(24.5) |  |
| **WHO score – interpretation** |  |  |  | <0.001 |
| > 50: Lower risk of depression | 416(97.4) | 2,394(76.7) | 2,810(79.2) |  |
| 29-50: 'Screening diagnosis' of depression | 9(2.1) | 547(17.5) | 556(15.7) |  |
| ≤ 28: Higher risk of depression | 2(0.5) | 181(5.8) | 183(5.2) |  |

*Individuals who answered all three follow-ups and the behavioral questionnaire were included in this analysis. Persistently favorable self-rated health was defined as individuals who reported very good self-rated health at all three follow-ups (2021, 2022 and 2023)

**References**

1. Idler EL, Benyamini Y. Self-rated health and mortality: a review of twenty-seven community studies. J Health Soc Behav. 1997 Mar;38(1):21-37. PMID: 9097506.

2. Mutz J, Lewis CM. Cross-classification between self-rated health and health status: longitudinal analyses of all-cause mortality and leading causes of death in the UK. Sci Rep. 2022 Jan 10;12(1):459. doi: 10.1038/s41598-021-04016-x. PMID: 35013388; PMCID: PMC8748682.

3. Jylhä M. What is self-rated health and why does it predict mortality? Towards a unified conceptual model. Soc Sci Med. 2009 Aug;69(3):307-16. doi: 10.1016/j.socscimed.2009.05.013. Epub 2009 Jun 10. PMID: 19520474.

4. Benjamins MR, Hummer RA, Eberstein IW, Nam CB. Self-reported health and adult mortality risk: an analysis of cause-specific mortality. Soc Sci Med. 2004 Sep;59(6):1297-306. doi: 10.1016/j.socscimed.2003.01.001. PMID: 15210100.

5. Miller TR, Wolinsky FD. Self-rated health trajectories and mortality among older adults. J Gerontol B Psychol Sci Soc Sci. 2007 Jan;62(1):S22-7. doi: 10.1093/geronb/62.1.s22. PMID: 17284562; PMCID: PMC2104470.

6. DeSalvo KB, Bloser N, Reynolds K, He J, Muntner P. Mortality prediction with a single general self-rated health question. A meta-analysis. J Gen Intern Med. 2006 Mar;21(3):267-75. doi: 10.1111/j.1525-1497.2005.00291.x. Epub 2005 Dec 7. PMID: 16336622; PMCID: PMC1828094.

7. Lekander M, Elofsson S, Neve IM, Hansson LO, Undén AL. Self-rated health is related to levels of circulating cytokines. Psychosom Med. 2004 Jul-Aug;66(4):559-63. doi: 10.1097/01.psy.0000130491.95823.94. PMID: 15272103.

8. Undén AL, Andréasson A, Elofsson S, Brismar K, Mathsson L, Rönnelid J, Lekander M. Inflammatory cytokines, behaviour and age as determinants of self-rated health in women. Clin Sci (Lond). 2007 Jun;112(6):363-73. doi: 10.1042/CS20060128. PMID: 17094770.

9. Halford C, Anderzén I, Arnetz B. Endocrine measures of stress and self-rated health: a longitudinal study. J Psychosom Res. 2003 Oct;55(4):317-20. doi: 10.1016/s0022-3999(02)00634-7. PMID: 14507542.

10. Nakata A, Takahashi M, Otsuka Y, Swanson NG. Is self-rated health associated with blood immune markers in healthy individuals? Int J Behav Med. 2010 Sep;17(3):234-42. doi: 10.1007/s12529-010-9102-0. PMID: 20512441.

11. Jylhä M, Guralnik JM, Ferrucci L, Jokela J, Heikkinen E. Is self-rated health comparable across cultures and genders? J Gerontol B Psychol Sci Soc Sci. 1998 May;53(3):S144-52. doi: 10.1093/geronb/53b.3.s144. PMID: 9602839.

12. Kaplan G, Baron-Epel O. What lies behind the subjective evaluation of health status? Soc Sci Med. 2003 Apr;56(8):1669-76. doi: 10.1016/s0277-9536(02)00179-x. PMID: 12639584.

13. Idler EL. Discussion: Gender Differences in Self-Rated Health, in Mortality, and in the Relationship Between the Two, The Gerontologist, Volume 43, Issue 3, June 2003, Pages 372–375, https://doi.org/10.1093/geront/43.3.372.

14. Dowd JB, Zajacova A. Does self-rated health mean the same thing across socioeconomic groups? Evidence from biomarker data. Ann Epidemiol. 2010 Oct;20(10):743-9. doi: 10.1016/j.annepidem.2010.06.007. PMID: 20816313; PMCID: PMC4845753.

15. Subramanian SV, Kim D, Kawachi I. Covariation in the socioeconomic determinants of self rated health and happiness: a multivariate multilevel analysis of individuals and communities in the USA. J Epidemiol Community Health. 2005 Aug;59(8):664-9. doi: 10.1136/jech.2004.025742. PMID: 16020643; PMCID: PMC1733107.

16. Sayón-Orea C, Santiago S, Bes-Rastrollo M, Martínez-González MA, Pastor MR, Moreno-Aliaga MJ, Tur JA, Garcia A, Martínez JA. Determinants of Self-Rated Health Perception in a Sample of a Physically Active Population: PLENUFAR VI Study. Int J Environ Res Public Health. 2018 Sep 25;15(10):2104. doi: 10.3390/ijerph15102104. PMID: 30257464; PMCID: PMC6210315.

17. Southwick SM, Bonanno GA, Masten AS, Panter-Brick C, Yehuda R. Resilience definitions, theory, and challenges: interdisciplinary perspectives. Eur J Psychotraumatol. 2014 Oct 1;5. doi: 10.3402/ejpt.v5.25338. PMID: 25317257; PMCID: PMC4185134.

18. Babić R, Babić M, Rastović P, Ćurlin M, Šimić J, Mandić K, Pavlović K. Resilience in Health and Illness. Psychiatr Danub. 2020 Sep;32(Suppl 2):226-232. PMID: 32970640.

19. Linnemann P, Berger K, Teismann H. Associations Between Outcome Resilience and Sociodemographic Factors, Childhood Trauma, Personality Dimensions and Self-Rated Health in Middle-Aged Adults. Int J Behav Med. 2022 Dec;29(6):796-806. doi: 10.1007/s12529-022-10061-1. Epub 2022 Mar 4. PMID: 35246825; PMCID: PMC9684253.

20. Wattick RA, Hagedorn RL, Olfert MD. Impact of resilience on college student mental health during COVID-19. J Am Coll Health. 2023 Oct;71(7):2184-2191. doi: 10.1080/07448481.2021.1965145. Epub 2021 Aug 27. PMID: 34448676.

21. Lau SYZ, Guerra RO, Barbosa JFS, Phillips SP. Impact of resilience on health in older adults: a cross-sectional analysis from the International Mobility in Aging Study (IMIAS). BMJ Open. 2018 Nov 28;8(11):e023779. doi: 10.1136/bmjopen-2018-023779. PMID: 30498045; PMCID: PMC6278794.

22. Šolcová I, Kebza V, Kodl M, Kernová V. Self-reported Health Status Predicting Resilience and Burnout in Longitudinal Study. Cent Eur J Public Health. 2017 Sep;25(3):222-227. doi: 10.21101/cejph.a4840. PMID: 29022682.

23. Atienza-González FL, Martínez N, Silva C. Life Satisfaction and Self-rated Health in Adolescents: The Relationships between them and the Role of Gender and Age. Span J Psychol. 2020 May 21;23:e4. doi: 10.1017/SJP.2020.10. PMID: 32436483.

24. Kim ES, Delaney SW, Tay L, Chen Y, Diener ED, Vanderweele TJ. Life Satisfaction and Subsequent Physical, Behavioral, and Psychosocial Health in Older Adults. Milbank Q. 2021 Mar;99(1):209-239. doi: 10.1111/1468-0009.12497. Epub 2021 Feb 2. PMID: 33528047; PMCID: PMC7984669.

25. Qazi SL, Koivumaa-Honkanen H, Rikkonen T, Sund R, Kröger H, Isanejad M, Sirola J. Physical capacity, subjective health, and life satisfaction in older women: a 10-year follow-up study. BMC Geriatr. 2021 Nov 23;21(1):658. doi: 10.1186/s12877-021-02605-z. PMID: 34814850; PMCID: PMC8609741.

26. Shi M, Liu L, Sun X, Wang L. Associations between symptoms of attention-deficit/ hyperactivity disorder and life satisfaction in medical students: the mediating effect of resilience. BMC Med Educ. 2018 Jul 13;18(1):164. doi: 10.1186/s12909-018-1261-8. PMID: 30005708; PMCID: PMC6043958.

27. Lüdecke D, von dem Knesebeck O. Worsened self-rated health in the course of the COVID-19 pandemic among older adults in Europe. Eur J Public Health. 2023 Aug 11:ckad143. doi: 10.1093/eurpub/ckad143. Epub ahead of print. PMID: 37568252.

28. de Boer WIJ, Mierau JO, Schoemaker J, Viluma L, Koning RH; Lifelines Corona Research Initiative. The impact of the Covid-19 crisis on socioeconomic differences in physical activity behavior: Evidence from the Lifelines COVID-19 cohort study. Prev Med. 2021 Dec;153:106823. doi: 10.1016/j.ypmed.2021.106823. Epub 2021 Oct 5. PMID: 34624391; PMCID: PMC8496996.

29. Arpino B, Pasqualini M, Bordone V. Physically distant but socially close? Changes in non-physical intergenerational contacts at the onset of the COVID-19 pandemic among older people in France, Italy and Spain. Eur J Ageing. 2021 Apr 26;18(2):185-194. doi: 10.1007/s10433-021-00621-x. PMID: 33935612; PMCID: PMC8074698.

30. Nehme M, Braillard O, Alcoba G, Aebischer Perone S, Courvoisier D, Chappuis F, Guessous I; COVICARE TEAM. COVID-19 Symptoms: Longitudinal Evolution and Persistence in Outpatient Settings. Ann Intern Med. 2021 May;174(5):723-725. doi: 10.7326/M20-5926. Epub 2020 Dec 8. PMID: 33284676; PMCID: PMC7741180.

31. Nehme M, Braillard O, Chappuis F, Courvoisier DS, Guessous I. Prevalence of Symptoms More Than Seven Months After Diagnosis of Symptomatic COVID-19 in an Outpatient Setting. Ann Intern Med. 2021 Jul 6. doi: 10.7326/M21-0878. Epub ahead of print. PMID: 34224254.

32. Baysson, H., Pennacchio, F., Wisniak, A., Zaballa, M.-E., Pullen, N., Collombet, P., Lorthe, E., Joost, S., Balavoine, J.-F., Bachmann, D., Azman, A.S., Pittet, D., Chappuis, F., Kherad, O., Kaiser, L., Guessous, I., Stringhini, S., Group, on behalf of the S.C. study, 2022. The Specchio-COVID19 cohort study: a longitudinal follow-up of SARS-CoV-2 serosurvey participants in the canton of Geneva, Switzerland (Study protocol). BMJ Open.

33. Stringhini, S., Zaballa, M.-E., Perez-Saez, J., Pullen, N., de Mestral, C., Picazio, A., Pennacchio, F., Wisniak, A., Richard, A., Baysson, H., Loizeau, A., Balavoine, J.-F., Trono, D., Pittet, D., Posfay-Barbe, K., Flahault, A., Chappuis, F., Kherad, O., Vuilleumier, N., Kaiser, L., Azman, A.S., Guessous, I., Specchio-COVID19 Study Group, 2021a. Seroprevalence of anti-SARS-CoV-2 antibodies after the second pandemic peak. Lancet Infect Dis 21, 600–601. https://doi.org/10.1016/S1473-3099(21)00054-2.

34. Kocalevent RD, Berg L, Beutel ME, Hinz A, Zenger M, Härter M, Nater U, Brähler E. Social support in the general population: standardization of the Oslo social support scale (OSSS-3). BMC Psychol. 2018 Jul 17;6(1):31. doi: 10.1186/s40359-018-0249-9. PMID: 30016997; PMCID: PMC6050647.

35. Topp CW, Østergaard SD, Søndergaard S, Bech P. The WHO-5 Well-Being Index: a systematic review of the literature. Psychother Psychosom. 2015;84(3):167-76. doi: 10.1159/000376585. Epub 2015 Mar 28. PMID: 25831962.

36. van de Weijer MP, de Vries LP, Pelt DHM, Ligthart L, Willemsen G, Boomsma DI, de Geus E, Bartels M. Self-rated health when population health is challenged by the COVID-19 pandemic; a longitudinal study. Soc Sci Med. 2022 Aug;306:115156. doi: 10.1016/j.socscimed.2022.115156. Epub 2022 Jun 16. PMID: 35728461; PMCID: PMC9212653.

37. Linton SL, Leifheit KM, McGinty EE, Barry CL, Pollack CE. Association Between Housing Insecurity, Psychological Distress, and Self-rated Health Among US Adults During the COVID-19 Pandemic. JAMA Netw Open. 2021 Sep 1;4(9):e2127772. doi: 10.1001/jamanetworkopen.2021.27772. Erratum in: JAMA Netw Open. 2021 Nov 1;4(11):e2137473. PMID: 34591109; PMCID: PMC8485162.

38. Peters A, Rospleszcz S, Greiser KH, Dallavalle M, Berger K; Complete list of authors available under: Collaborators. The Impact of the COVID-19 Pandemic on Self-Reported Health. Dtsch Arztebl Int. 2020 Dec 11;117(50):861-867. doi: 10.3238/arztebl.2020.0861. PMID: 33295275; PMCID: PMC8025933.

39. Szwarcwald CL, Damacena GN, Barros MBA, Malta DC, Souza Júnior PRB, Azevedo LO, Machado ÍE, Lima MG, Romero D, Gomes CS, Werneck AO, Silva DRPD, Gracie R, Pina MF. Factors affecting Brazilians’ self-rated health during the COVID-19 pandemic. Cad Saude Publica. 2021 Apr 30;37(3):e00182720. doi: 10.1590/0102-311X00182720. PMID: 33950075.

40. Abu-Omar K, Rütten A, Robine JM. Self-rated health and physical activity in the European Union. Soz Praventivmed. 2004;49(4):235-42. doi: 10.1007/s00038-004-3107-x. PMID: 15357524.

41. Beyer AK, Wolff JK, Warner LM, Schüz B, Wurm S. The role of physical activity in the relationship between self-perceptions of ageing and self-rated health in older adults. Psychol Health. 2015;30(6):671-85. doi: 10.1080/08870446.2015.1014370. Epub 2015 Feb 27. PMID: 25720739.

42. Shirom A, Toker S, Berliner S, Shapira I, Melamed S. The effects of physical fitness and feeling vigorous on self-rated health. Health Psychol. 2008 Sep;27(5):567-75. doi: 10.1037/0278-6133.27.5.567. PMID: 18823183.

43. Magee CA, Caputi P, Iverson DC. Patterns of health behaviours predict obesity in Australian children. J Paediatr Child Health. 2013 Apr;49(4):291-6. doi: 10.1111/jpc.12163. PMID: 23574555.

44. Nie X, Li Y, Li C, Wu J, Li L. The Association Between Health Literacy and Self-rated Health Among Residents of China Aged 15-69 Years. Am J Prev Med. 2021 Apr;60(4):569-578. doi: 10.1016/j.amepre.2020.05.032. Epub 2021 Feb 12. PMID: 33583676.

45. Herman KM, Hopman WM, Sabiston CM. Physical activity, screen time and self-rated health and mental health in Canadian adolescents. Prev Med. 2015 Apr;73:112-6. doi: 10.1016/j.ypmed.2015.01.030. Epub 2015 Feb 4. PMID: 25660484.

46. Frange C, de Queiroz SS, da Silva Prado JM, Tufik S, de Mello MT. The impact of sleep duration on self-rated health. Sleep Sci. 2014 Jun;7(2):107-13. doi: 10.1016/j.slsci.2014.09.006. Epub 2014 Sep 16. PMID: 26483912; PMCID: PMC4521644.

47. Štefan L, Juranko D, Prosoli R, Barić R, Sporiš G. Self-Reported Sleep Duration and Self-Rated Health in Young Adults. J Clin Sleep Med. 2017 Jul 15;13(7):899-904. doi: 10.5664/jcsm.6662. PMID: 28502281; PMCID: PMC5482581.

48. Buysse DJ, Angst J, Gamma A, Ajdacic V, Eich D, Rössler W. Prevalence, course, and comorbidity of insomnia and depression in young adults. Sleep. 2008 Apr;31(4):473-80. doi: 10.1093/sleep/31.4.473. PMID: 18457234; PMCID: PMC2279748.

49. Glozier N, Martiniuk A, Patton G, Ivers R, Li Q, Hickie I, Senserrick T, Woodward M, Norton R, Stevenson M. Short sleep duration in prevalent and persistent psychological distress in young adults: the DRIVE study. Sleep. 2010 Sep;33(9):1139-45. doi: 10.1093/sleep/33.9.1139. PMID: 20857859; PMCID: PMC2938854.

50. Zack MM, Moriarty DG, Stroup DF, Ford ES, Mokdad AH. Worsening trends in adult health-related quality of life and self-rated health-United States, 1993-2001. Public Health Rep. 2004 Sep-Oct;119(5):493-505. doi: 10.1016/j.phr.2004.07.007. PMID: 15313113; PMCID: PMC1497661.

51. Svence G, Majors M. Correlation of well-being with resilience and age. Problems of psychology in the 21st century. Vol 9, No.1, 2015.

52. Fayers PM, Sprangers MA. Understanding self-rated health. Lancet. 2002 Jan 19;359(9302):187-8. doi: 10.1016/S0140-6736(02)07466-4. PMID: 11812551.

53. Barsky AJ, Peekna HM, Borus JF. Somatic symptom reporting in women and men. J Gen Intern Med. 2001 Apr;16(4):266-75. doi: 10.1046/j.1525-1497.2001.00229.x. PMID: 11318929; PMCID: PMC1495200.

Table S1. Baseline characteristics of participants and non-participants

|  |  | **2021**  **(n=7,006)** | **2022**  **(n=6,704)** | **2023**  **(n=5,519)** | **Participants**  **to all 3 follow-ups**  **(n=3,888)** | **Non-**  **Participants**  **(n=4,184)** | **P-value** |
| --- | --- | --- | --- | --- | --- | --- | --- |
|  |  | N(%) | N(%) | N(%) | N(%) | N(%) |  |
| **Age groups** |  |  |  |  |  |  | <0.001 |
| under 25 |  | 241(3.4) | 164(2.4) | 134(2.4) | 61(1.6) | 422(10.1) |  |
| between 25-40 |  | 1,395(19.9) | 1,162(17.3) | 918(16.6) | 545(14.0) | 1,422(34.0) |  |
| between 40-65 |  | 4,357(62.2) | 4,237(63.2) | 3,473(62.9) | 2.506(64.4) | 1,997(47.7) |  |
| 65 and above |  | 1,013(14.5) | 1,141(17.0) | 994(18.0) | 776(20.0) | 343(8.2) |  |
| **Sex** |  |  |  |  |  |  | 0.977 |
| Male |  | 2,942(42) | 2,728(40.7) | 2,238(40.6) | 1,604(41.3) | 1,822(43.5) |  |
| Female |  | 4,036(57.6) | 3,955(59) | 3,262(59.1) | 2,271(58.4) | 2,343(56.0) |  |
| Other |  | 28(0.4) | 21(0.3) | 19(0.3) | 13(0.3) | 20(0.5) |  |
| **Education** |  |  |  |  |  |  | <0.001 |
| Primary |  | 249(3.6) | 258(3.8) | 179(3.2) | 112(2.9) | 364(8.7) |  |
| Secondary |  | 2,207(31.5) | 2,105(31.4) | 1,698(30.8) | 1,234(31.7) | 1,272(30.4) |  |
| Tertiary |  | 4,545(64.9) | 4,327(64.6) | 3,634(65.9) | 2,538(65.3) | 2,531(60.5) |  |
| Other |  | 5(0.1) | 13(0.2) | 6(0.1) | 4(0.1) | 15(0.4) |  |
| **Work situation** |  |  |  |  |  |  | <0.001 |
| Salaried |  | 4,765(68.0) | 4,392(65.5) | 3,533(64) | 2,474(63.6) | 2,757(66) |  |
| Freelance/sole trader |  | 475(6.8) | 459(6.8) | 386(7) | 287(7.4) | 239(5.7) |  |
| Retired |  | 1,058(15.1) | 1,207(18) | 1,057(19.2) | 807(20.8) | 354(8.5) |  |
| Unemployed |  | 165(2.4) | 151(2.3) | 135(2.4) | 75(1.9) | 166(4) |  |
| Other economically inactive |  | 542(7.7) | 494(7.4) | 406(7.4) | 244(6.3) | 662(15.8) |  |
| **Profession** |  |  |  |  |  |  | <0.001 |
| Blue collar workers |  | 637(9.5) | 594(9.3) | 427(8.1) | 298(7.7) | 564(15.8) |  |
| Lower grade white collar workers |  | 1,732(26) | 1,701(26.7) | 1,388(26.4) | 999(25.7) | 856(23.9) |  |
| Higher-grade white collar workers |  | 1,978(29.6) | 1,841(28.9) | 1,553(29.5) | 1,116(28.7) | 948(26.5) |  |
| Professional-Managers |  | 2,235(33.5) | 2,124(33.3) | 1,792(34.1) | 51(1.3) | 1,114(31.1) |  |
| Independent workers |  | 90(1.3) | 121(1.9) | 100(1.9) | 1,294(33.3) | 98(2.7) |  |
| **Nationality** |  |  |  |  |  |  |  |
| Swiss nationals |  | 5,544(79.1) | 5,337(79.6) | 4,473(81.0) | 3,241(83.4) | 2,635(63.0) | <0.001 |
| Non-Swiss nationals |  | 1,462(20.9) | 1,366(20.4) | 1,046(19.0) | 647(16.6) | 1,545(37.0) |  |
| **Living status** |  |  |  |  | 127(3.3) |  | <0.001 |
| Single |  | 1,029(14.7) | 1,021(15.2) | 841(15.2) | 635(16.3) | 505(12.1) |  |
| Single parent |  | 484(6.9) | 443(6.6) | 385(7) | 249(6.4) | 293(7) |  |
| With partner and kids |  | 3,119(44.5) | 2,975(44.4) | 2,381(43.2) |  | 2,118(50.8) |  |
| With partner, without kids |  | 1,877(26.8) | 1,861(27.8) | 1,587(28.8) | 1,594(41) | 576(13.8) |  |
| Cohabitation |  | 497(7.1) | 401(6) | 323(5.9) | 1,410(36.3) | 681(16.3) |  |
| **Household income** |  |  |  |  |  |  | <0.001 |
| Low |  | 1,048(15.0) | 982(14.7) | 753(13.7) | 497(12.8) | 858(20.7) |  |
| Middle |  | 3,768(53.8) | 3,593(53.6) | 3,044(55.2) | 2,199(56.6) | 1,765(42.5) |  |
| High |  | 960(13.7) | 955(14.3) | 795(14.4) | 574(14.8) | 491(11.8) |  |
| Don't know/don't wish to answer |  | 1,229(17.5) | 1,168(17.4) | 924(16.8) | 618(15.9) | 1,478(23.6) |  |
| **Pre-existing Comorbidities** |  | 1,499(21.4) | 1,520(22.7) | 1,262(22.9) | 893(23.0) | 797(19.0) | <0.001 |
| **Pre-existing mental health condition** |  | 147(2.1) | 157(2.3) | 125(2.3) | 77(2.0) | 101(2.4) | 0.186 |

Table S2. Associations between persistently favorable self-rated health and age, sex, socio-economic determinants and comorbidities.

|  |  | OR | 95%CI | P-value |  | aOR | 95%CI | P-value |
| --- | --- | --- | --- | --- | --- | --- | --- | --- |
| Age groups | under 25 | - | - | - |  | - | - | - |
|  | 25-39 | 0.59 | [0.29-1.20] | 0.147 |  | 0.50 | [0.07-3.44] | 0.478 |
|  | 40-64 | 0.63 | [0.32-1.23] | 0.176 |  | 0.48 | [0.07-3.28] | 0.452 |
|  | 65 and above | 0.61 | [0.31-1.22] | 0.161 |  | 0.43 | [0.06-3.24] | 0.410 |
| Sex* | Male | - | - | - |  | - | - | - |
|  | Female | 0.78 | [0.64-0.94] | 0.011 |  | 0.83 | [0.64-1.07] | 0.152 |
| Education | Primary | - | - | - |  | - | - | - |
|  | Secondary | 1.52 | [0.75-3.08] | 0.240 |  | 1.47 | [0.53-4.07] | 0.454 |
|  | Tertiary | 1.64 | [0.82-3.27] | 0.161 |  | 1.23 | [0.44-3.42] | 0.695 |
| Work situation | Retired | - | - | - |  | - | - | - |
|  | Salaried | 1.04 | [0.81-1.33] | 0.763 |  | 1.00 | [0.55-1.82] | 0.991 |
|  | Freelance | 1.34 | [0.91-1.97] | 0.144 |  | 1.14 | [0.59-2.21] | 0.693 |
|  | Unemployed | 0.66 | [0.28-1.56] | 0.344 |  | 0.56 | [0.14-2.2] | 0.408 |
|  | Other | 1.06 | [0.69-1.65] | 0.784 |  | 0.56 | [0.21-1.48] | 0.240 |
| Profession | Blue collar workers | - | - | - |  | - | - | - |
|  | Lower | 1.21 | [0.791.84] | 0.381 |  | 1.44 | [0.81-2.57] | 0.217 |
|  | Higher-grade | 1.09 | [0.71-1.66] | 0.700 |  | 1.25 | [0.69-2.26] | 0.468 |
|  | Professional-Managers | 1.46 | [0.97-2.20] | 0.068 |  | 1.32 | [0.72-2.43] | 0.365 |
|  | Independent | 0.97 | [0.36-2.63] | 0.954 |  | 0.47 | [0.12-1.91] | 0.294 |
| Nationality | Swiss nationals | - | - | - |  | - | - | - |
|  | Non-Swiss nationals | 0.92 | [0.71-1.20] | 0.549 |  | 1.00 | [0.74-1.34] | 0.982 |
| Living status | Single | - | - | - |  | - | - | - |
|  | Single parent | 1.13 | [0.72-1.77] | 0.596 |  | 1.18 | [0.67-2.08] | 0.555 |
|  | With partners and kids | 1.12 | [0.84-1.49] | 0.440 |  | 1.01 | [0.69-1.47] | 0.960 |
|  | With partner without kids | 1.46 | [0.91-2.32] | 0.113 |  | 0.81 | [0.56-1.18] | 0.267 |
|  | Cohabitation | 1.06 | [0.78-1.43] | 0.705 |  | 1.35 | [0.63-2.88] | 0.434 |
| Household income | Low | - | - | - |  | - | - | - |
|  | Middle | 1.31 | [0.94-1.83] | 0.104 |  | 1.47 | [0.97-2.23] | 0.068 |
|  | High | 2.22 | [1.53-3.21] | <0.001 |  | 2.03 | [1.24-3.33] | 0.005 |
| Pre-existing comorbidities | Yes | 0.29 | [0.21-0.40] | <0.001 |  | 0.29 | [0.19-0.44] | <0.001 |
| Pre-existing mental health condition | Yes | 0.39 | [0.14-1.08] | 0.069 |  | 0.74 | [0.2-2.64] | 0.638 |
|  |  |  |  |  |  |  |  |  |

OR: Odds ratio

aOR: adjusted odds ratio, adjusted for age groups, sex, education, work situation, profession, living status, household income, pre-existing comorbidities, pre-existing mental health condition, alcohol, smoking, social support, and mental health status.

95%CI: 95% confidence interval

Sex was defined as male, female or other however due to small observations for other (n= 13), the analysis could not be conducted

Table S3. Distribution of health behavior by age groups

|  | **under 25** | **between 25 and 40** | **between 40 and 65** | **65 and above** | **Total** | **P-value** |
| --- | --- | --- | --- | --- | --- | --- |
|  | N(%) | N(%) | N(%) | N(%) | N(%) |  |
| **Alcohol** |  |  |  |  |  | <0.001 |
| Never | 7(11.5) | 61(11.2) | 216(8.6) | 58(7.5) | 342(8.8) |  |
| Occasionally | 36(59) | 217(39.8) | 910(36.3) | 234(30.2) | 1,397(35.9) |  |
| Once a week | 8(13.1) | 73(13.4) | 292(11.7) | 62(8) | 435(11.2) |  |
| 2-3 times per week | 8(13.1) | 174(31.9) | 823(32.8) | 236(30.4) | 1,241(31.9) |  |
| Daily | 2(3.3) | 18(3.3) | 243(9.7) | 157(20.2) | 420(10.8) |  |
| Several times a day | 0(0) | 2(0.4) | 22(0.9) | 29(3.7) | 53(1.4) |  |
| **Smoking** |  |  |  |  |  | <0.001 |
| Never smokers | 9(14.8) | 90(16.5) | 396(15.8) | 81(10.4) | 576(14.8) |  |
| Former smokers | 2(3.3) | 124(22.8) | 735(29.3) | 338(43.6) | 1,199(30.8) |  |
| Current smokers | 50(82) | 331(60.7) | 1,375(54.9) | 357(46) | 2,113(54.3) |  |
| **Exercise** |  |  |  |  |  | <0.001 |
| Never | 11(18) | 89(16.3) | 571(22.8) | 244(31.4) | 915(23.5) |  |
| Occasionally | 12(19.7) | 140(25.7) | 615(24.5) | 198(25.5) | 965(24.8) |  |
| Once a week | 16(26.2) | 141(25.9) | 603(24.1) | 136(17.5) | 896(23) |  |
| 2-3 times a week | 15(24.6) | 133(24.4) | 548(21.9) | 133(17.1) | 829(21.3) |  |
| 3-4 times a week | 5(8.2) | 29(5.3) | 119(4.7) | 32(4.1) | 185(4.8) |  |
| Daily | 2(3.3) | 13(2.4) | 50(2) | 33(4.3) | 98(2.5) |  |
| **Exercise during pandemic** |  |  |  |  |  | <0.001 |
| Decreased | 12(23.5) | 56(11.9) | 194(8.5) | 29(3.9) | 291(8.2) |  |
| Slightly decreased | 8(15.7) | 91(19.3) | 309(13.6) | 98(13.1) | 506(14.3) |  |
| Did not change | 18(35.3) | 194(41.1) | 1,237(54.3) | 467(62.3) | 1,916(54) |  |
| Slightly increased | 4(7.8) | 75(15.9) | 342(15) | 120(16) | 541(15.2) |  |
| Increased | 9(17.6) | 56(11.9) | 195(8.6) | 35(4.7) | 295(8.3) |  |
| **Healthy diet habits** |  |  |  |  |  | <0.001 |
| No | 2(3.9) | 7(1.5) | 16(0.7) | 3(0.4) | 28(0.8) |  |
| Mostly no | 7(13.7) | 55(11.7) | 158(6.9) | 21(2.8) | 241(6.8) |  |
| Yes | 12(23.5) | 100(21.2) | 581(25.5) | 289(38.6) | 982(27.7) |  |
| Mostly yes | 30(58.8) | 305(64.6) | 1,487(65.3) | 431(57.5) | 2,253(63.5) |  |
| Do not know | 0(0) | 5(1.1) | 35(1.5) | 5(0.7) | 45(1.3) |  |
| **Diet habits during pandemic** |  |  |  |  |  | <0.001 |
| Improved | 6(11.8) | 34(7.2) | 130(5.7) | 15(2) | 185(5.2) |  |
| Slighly improved | 3(5.9) | 66(14) | 284(12.5) | 66(8.8) | 419(11.8) |  |
| Worsened | 3(5.9) | 12(2.5) | 23(1) | 0(0) | 38(1.1) |  |
| Slightly worsened | 10(19.6) | 50(10.6) | 138(6.1) | 17(2.3) | 215(6.1) |  |
| Did not change | 29(56.9) | 310(65.7) | 1,702(74.7) | 651(86.9) | 2,692(75.9) |  |
| **Screen use** |  |  |  |  |  | <0.001 |
| Never | 0(0.0) | 1(0.2) | 12(0.5) | 3(0.4) | 16(0.5) |  |
| < 1 hour per day | 3(5.9) | 41(8.7) | 358(15.7) | 134(17.9) | 536(15.1) |  |
| 1 hour to <2 hours per day | 9(17.6) | 154(32.6) | 863(37.9) | 249(33.2) | 1,275(35.9) |  |
| 2 hours to <4 hours per day | 26(51.0) | 204(43.2) | 832(36.5) | 272(36.3) | 1,334(37.6) |  |
| 4 hours to <8 hours per day | 10(19.6) | 51(10.8) | 156(6.9) | 85(11.3) | 302(8.5) |  |
| ≥ 8 hours per day | 3(5.9) | 21(4.4) | 56(2.5) | 6(0.8) | 86(2.4) |  |
| **Screen habits during pandemic** |  |  |  |  |  | <0.001 |
| Increased | 13(25.5) | 66(14) | 258(11.3) | 45(6) | 382(10.8) |  |
| Slightly increased | 16(31.4) | 107(22.7) | 517(22.7) | 148(19.8) | 788(22.2) |  |
| Decreased | 1(2.0) | 8(1.7) | 15(0.7) | 10(1.3) | 34(1.0) |  |
| Slightly decreased | 3(5.9) | 24(5.1) | 60(2.6) | 13(1.7) | 100(2.8) |  |
| Did not change | 18(35.3) | 267(56.6) | 1,427(62.7) | 533(71.2) | 2,245(63.3) |  |
| **Sleep time** |  |  |  |  |  | <0.001 |
| < 5 hours | 0(0) | 5(1.1) | 38(1.7) | 9(1.2) | 52(1.5) |  |
| 5-6 hours | 2(3.9) | 61(12.9) | 279(12.3) | 90(12) | 432(12.2) |  |
| 6-7 hours | 11(21.6) | 159(33.7) | 798(35) | 225(30) | 1,193(33.6) |  |
| 7-8 hours | 23(45.1) | 197(41.7) | 950(41.7) | 306(40.9) | 1,476(41.6) |  |
| 8-9 hours | 10(19.6) | 46(9.7) | 190(8.3) | 107(14.3) | 353(9.9) |  |
| 9-10 hours | 4(7.8) | 4(0.8) | 18(0.8) | 9(1.2) | 35(1.0) |  |
| ≥ 10 hours | 1(2.0) | 0(0.0) | 4(0.2) | 3(0.4) | 8(0.2) |  |
| **Sleep quality** |  |  |  |  |  | <0.001 |
| Very good | 7(13.7) | 49(10.4) | 306(13.4) | 159(21.2) | 521(14.7) |  |
| Good | 34(66.7) | 266(56.4) | 1,369(60.1) | 479(64) | 2,148(60.5) |  |
| Poor | 8(15.7) | 143(30.3) | 554(24.3) | 103(13.8) | 808(22.8) |  |
| Very poor | 2(3.9) | 14(3) | 48(2.1) | 8(1.1) | 72(2) |  |
| **Sleep difficulties** |  |  |  |  |  | 0.037 |
| Never | 13(25.5) | 99(21) | 455(20) | 168(22.4) | 735(20.7) |  |
| Sometimes | 28(54.9) | 252(53.4) | 1,231(54.1) | 439(58.6) | 1,950(54.9) |  |
| Often | 8(15.7) | 94(19.9) | 476(20.9) | 118(15.8) | 696(19.6) |  |
| All the time | 2(3.9) | 27(5.7) | 115(5.1) | 24(3.2) | 168(4.7) |  |
| **Sleep habits during pandemic** |  |  |  |  |  | <0.001 |
| Improved | 1(2.0) | 11(2.3) | 38(1.7) | 3(0.4) | 53(1.5) |  |
| Slightly improved | 2(3.9) | 26(5.5) | 74(3.2) | 16(2.1) | 118(3.3) |  |
| Worsened | 2(3.9) | 33(7) | 100(4.4) | 20(2.7) | 155(4.4) |  |
| Slightly worsened | 10(19.6) | 103(21.8) | 450(19.8) | 79(10.5) | 642(18.1) |  |
| Did not change | 36(70.6) | 299(63.3) | 1,615(70.9) | 631(84.2) | 2,581(72.7) |  |
| **Isolated** |  |  |  |  |  | <0.001 |
| Never | 33(54.1) | 276(50.6) | 1,523(60.8) | 482(62.1) | 2,314(59.5) |  |
| Rarely | 15(24.6) | 137(25.1) | 574(22.9) | 192(24.7) | 918(23.6) |  |
| Sometimes | 8(13.1) | 103(18.9) | 350(14) | 93(12) | 554(14.2) |  |
| Most of the time | 5(8.2) | 26(4.8) | 49(2) | 9(1.2) | 89(2.3) |  |
| All the time | 0(0) | 3(0.6) | 10(0.4) | 0(0) | 13(0.3) |  |
| **Oslo score – interpretation** |  |  |  |  |  | 0.360 |
| ≤ 8: Poor social support | 6(9.8) | 100(18.3) | 399(15.9) | 136(17.5) | 641(16.5) |  |
| 9-11: Moderate social support | 42(68.9) | 305(56.0) | 1,479(59.0) | 457(58.9) | 2,283(58.7) |  |
| ≥ 12: High social support | 13(21.3) | 140(25.7) | 628(25.1) | 183(23.6) | 964(24.8) |  |
| **WHO score - interpretation** |  |  |  |  |  | <0.001 |
| > 50: Lower risk of depression | 45(73.8) | 407(74.7) | 1,951(77.9) | 660(85.1) | 3,063(78.8) |  |
| 29-50: 'Screening diagnosis' of depression | 14(23.0) | 98(18.0) | 418(16.7) | 90(11.6) | 620(15.9) |  |
| ≤ 28: Higher risk of depression | 2(3.3) | 40(7.3) | 137(5.5) | 26(3.4) | 205(5.3) |  |

Table S4. Distribution of health behavior by sex

|  | **Male** | **Female** | **Other** | **Total** | **P-value** |
| --- | --- | --- | --- | --- | --- |
|  | N(%) | N(%) | N(%) | N(%) |  |
| **Alcohol** |  |  |  |  | <0.001 |
| Never | 101(6.3) | 241(10.6) | 0(0.0) | 342(8.8) |  |
| Occasionally | 451(28.1) | 939(41.3) | 7(53.8) | 1,397(35.9) |  |
| Once a week | 182(11.3) | 250(11) | 3(23.1) | 435(11.2) |  |
| 2-3 times per week | 601(37.5) | 637(28) | 3(23.1) | 1,241(31.9) |  |
| Daily | 235(14.7) | 185(8.1) | 0(0.0) | 420(10.8) |  |
| Several times a day | 34(2.1) | 19(0.8) | 0(0.0) | 53(1.4) |  |
| **Smoking** |  |  |  |  | 0.208 |
| Never smokers | 239(14.9) | 337(14.8) | 0(0.0) | 576(14.8) |  |
| Former smokers | 507(31.6) | 690(30.4) | 2(15.4) | 1,199(30.8) |  |
| Current smokers | 858(53.5) | 1,244(54.8) | 11(84.6) | 2,113(54.3) |  |
| **Exercise** |  |  |  |  | <0.001 |
| Never | 322(20.1) | 592(26.1) | 1(7.7) | 915(23.5) |  |
| Occasionally | 363(22.6) | 596(26.2) | 6(46.2) | 965(24.8) |  |
| Once a week | 369(23) | 525(23.1) | 2(15.4) | 896(23) |  |
| 2-3 times a week | 387(24.1) | 438(19.3) | 4(30.8) | 829(21.3) |  |
| 3-4 times a week | 109(6.8) | 76(3.3) | 0(0.0) | 185(4.8) |  |
| Daily | 54(3.4) | 44(1.9) | 0(0.0) | 98(2.5) |  |
| **Exercise during pandemic** |  |  |  |  | 0.048 |
| Decreased | 114(7.8) | 177(8.5) | 0(0.0) | 291(8.2) |  |
| Slightly decreased | 215(14.7) | 286(13.8) | 5(38.5) | 506(14.3) |  |
| Did not change | 814(55.8) | 1,095(52.7) | 7(53.8) | 1,916(54) |  |
| Slightly increased | 210(14.4) | 331(15.9) | 0(0) | 541(15.2) |  |
| Increased | 106(7.3) | 188(9.1) | 1(7.7) | 295(8.3) |  |
| **Healthy diet habits** |  |  |  |  | 0.080 |
| No | 13(0.9) | 15(0.7) | 0(0.0) | 28(0.8) |  |
| Mostly no | 114(7.8) | 127(6.1) | 0(0.0) | 241(6.8) |  |
| Yes | 374(25.6) | 603(29) | 5(38.5) | 982(27.7) |  |
| Mostly yes | 938(64.3) | 1,308(63) | 7(53.8) | 2,253(63.5) |  |
| Do not know | 20(1.4) | 24(1.2) | 1(7.7) | 45(1.3) |  |
| **Diet habits during pandemic** |  |  |  |  | 0.007 |
| Improved | 67(4.6) | 118(5.7) | 0(0.0) | 185(5.2) |  |
| Slighly improved | 186(12.7) | 233(11.2) | 0(0.0) | 419(11.8) |  |
| Worsened | 8(0.5) | 29(1.4) | 1(7.7) | 38(1.1) |  |
| Slightly worsened | 77(5.3) | 138(6.6) | 0(0.0) | 215(6.1) |  |
| Did not change | 1,121(76.8) | 1,559(75.1) | 12(92.3) | 2,692(75.9) |  |
| **Screen use** |  |  |  |  | 0.085 |
| Never | 8(0.5) | 8(0.4) | 0(0.0) | 16(0.5) |  |
| < 1 hour per day | 243(16.7) | 291(14) | 2(15.4) | 536(15.1) |  |
| 1 hour to <2 hours per day | 504(34.5) | 767(36.9) | 4(30.8) | 1,275(35.9) |  |
| 2 hours to <4 hours per day | 520(35.6) | 809(39) | 5(38.5) | 1,334(37.6) |  |
| 4 hours to <8 hours per day | 144(9.9) | 157(7.6) | 1(7.7) | 302(8.5) |  |
| ≥ 8 hours per day | 40(2.7) | 45(2.2) | 1(7.7) | 86(2.4) |  |
| **Screen habits during pandemic** |  |  |  |  | 0.016 |
| Increased | 125(8.6) | 255(12.3) | 2(15.4) | 382(10.8) |  |
| Slightly increased | 304(20.8) | 481(23.2) | 3(23.1) | 788(22.2) |  |
| Decreased | 16(1.1) | 18(0.9) | 0(0.0) | 34(1) |  |
| Slightly decreased | 42(2.9) | 58(2.8) | 0(0.0) | 100(2.8) |  |
| Did not change | 972(66.6) | 1,265(60.9) | 8(61.5) | 2,245(63.3) |  |
| **Sleep time** |  |  |  |  | 0.137 |
| < 5 hours | 25(1.7) | 27(1.3) | 0(0.0) | 52(1.5) |  |
| 5-6 hours | 190(13) | 241(11.6) | 1(7.7) | 432(12.2) |  |
| 6-7 hours | 508(34.8) | 683(32.9) | 2(15.4) | 1,193(33.6) |  |
| 7-8 hours | 587(40.2) | 882(42.5) | 7(53.8) | 1,476(41.6) |  |
| 8-9 hours | 138(9.5) | 213(10.3) | 2(15.4) | 353(9.9) |  |
| 9-10 hours | 9(0.6) | 25(1.2) | 1(7.7) | 35(1.0) |  |
| ≥ 10 hours | 2(0.1) | 6(0.3) | 0(0.0) | 8(0.2) |  |
| **Sleep quality** |  |  |  |  | 0.008 |
| Very good | 253(17.3) | 267(12.9) | 1(7.7) | 521(14.7) |  |
| Good | 849(58.2) | 1,288(62) | 11(84.6) | 2,148(60.5) |  |
| Poor | 330(22.6) | 477(23) | 1(7.7) | 808(22.8) |  |
| Very poor | 27(1.9) | 45(2.2) | 0(0.0) | 72(2.0) |  |
| **Sleep difficulties** |  |  |  |  | <0.001 |
| Never | 404(27.7) | 329(15.8) | 2(15.4) | 735(20.7) |  |
| Sometimes | 759(52) | 1,180(56.8) | 11(84.6) | 1,950(54.9) |  |
| Often | 236(16.2) | 460(22.1) | 0(0.0) | 696(19.6) |  |
| All the time | 60(4.1) | 108(5.2) | 0(0.0) | 168(4.7) |  |
| **Sleep habits during pandemic** |  |  |  |  |  |
| Improved | 18(1.2) | 35(1.7) | 0(0.0) | 53(1.5) |  |
| Slightly improved | 54(3.7) | 62(3) | 2(15.4) | 118(3.3) |  |
| Worsened | 56(3.8) | 99(4.8) | 0(0) | 155(4.4) |  |
| Slightly worsened | 243(16.7) | 398(19.2) | 1(7.7) | 642(18.1) |  |
| Did not change | 1,088(74.6) | 1,483(71.4) | 10(76.9) | 2,581(72.7) |  |
| **Isolated** |  |  |  |  | 0.010 |
| Never | 1,007(62.8) | 1,298(57.2) | 9(69.2) | 2,314(59.5) |  |
| Rarely | 365(22.8) | 551(24.3) | 2(15.4) | 918(23.6) |  |
| Sometimes | 192(12) | 360(15.9) | 2(15.4) | 554(14.2) |  |
| Most of the time | 32(2) | 57(2.5) | 0(0.0) | 89(2.3) |  |
| All the time | 8(0.5) | 5(0.2) | 0(0.0) | 13(0.3) |  |
| **Oslo score – interpretation** |  |  |  |  | 0.240 |
| ≤ 8: Poor social support | 269(16.8) | 370(16.3) | 2(15.4) | 641(16.5) |  |
| 9-11: Moderate social support | 968(60.3) | 1,307(57.6) | 8(61.5) | 2,283(58.7) |  |
| ≥ 12: High social support | 367(22.9) | 594(26.2) | 3(23.1) | 964(24.8) |  |
| **WHO score – interpretation** |  |  |  |  | <0.001 |
| > 50: Lower risk of depression | 1,325(82.6) | 1,726(76) | 12(92.3) | 3,063(78.8) |  |
| 29-50: 'Screening diagnosis' of depression | 211(13.2) | 408(18) | 1(7.7) | 620(15.9) |  |
| ≤ 28: Higher risk of depression | 68(4.2) | 137(6) | 0(0.0) | 205(5.3) |  |
